# *Paraholcoglossum* and *Tsiorchis*, Two New Orchid Genera Established by Molecular and Morphological Analyses of the *Holcoglossum* Alliance

**DOI:** 10.1371/journal.pone.0024864

**Published:** 2011-10-10

**Authors:** Zhong-Jian Liu, Li-Jun Chen, Sing-Chi Chen, Jing Cai, Wen-Chieh Tsai, Yu-Yun Hsiao, Wen-Hui Rao, Xue-Yong Ma, Guo-Qiang Zhang

**Affiliations:** 1 Shenzhen Key Laboratory for Orchid Conservation and Utilization, The National Orchid Conservation Center of China and The Orchid Conservation and Research Center of Shenzhen, Shenzhen, China; 2 The Center for Biotechnology and BioMedicine, Graduate School at Shenzhen, Tsinghua University, Shenzhen, China; 3 College of Forestry, South China Agricultural University, Guangzhou, China; 4 State Key Laboratory of Systematic and Evolutionary Botany, Institute of Botany, Chinese Academy of Sciences, Beijing, China; 5 Institute of Tropical Plant Sciences, and Orchid Research Center, National Cheng Kung University, Taiwan, China; 6 Department of Life Sciences, National Cheng Kung University, Taiwan, China; University of Melbourne, Australia

## Abstract

**Background:**

*Holcoglossum* is a small orchid genus of 12 species ranging from SW China to Thailand and NE India. Although molecular and morphological analyses have been performed to establish the phylogenetic relationships within this genus, the interspecific relations and its relations with allied genera, such as *Rhynchostylis*, *Aerides* and *Vanda*, remain unclear.

**Methodology/Principal Findings:**

In addition to morphological analysis, maximum parsimony, maximum likelihood, and Bayesian inference analyses were performed based on fragments of the nuclear ITS and chloroplast *trnL-F* and *matK* genes of 31 taxa (15 *Holcoglossum*, 14 *Aeridinae*, 2 outgroups) representing all major clades of the *Holcoglossum* alliance. The results suggest that *Holcoglossum* is triphyletic, comprising three clades: the *Holcoglossum* clade, its sister clade, and a distant clade more closely related to *Rhynchostylis*, *Aerides*, and *Vanda* than to the *Holcoglossum* clade. The *Holcoglossum* clade is further divided into three subclades; the genetic distances between these three subclades also support this delimitation. The molecular conclusion is consistent with their distinct morphological characters.

**Conclusions:**

We propose that the latter two clades comprise two new genera, *Paraholcoglossum* and *Tsiorchis*, and *Holcoglossum* clade divides into three sections. In addition, a new section, *Holcoglossum* sect. *Nujiangensia*, and a new species, *Holcoglossum linearifolium*, are proposed. Some new combinations are made, and a new scheme is provided for the classification of all species of *Holcoglossum*, *Paraholcoglossum*, and *Tsiorchis*.

## Introduction

*Holcoglossum* is a small genus of Orchidaceae (*Aeridinae*, *Vandeae*) comprising about 12 species ranging from China to Vietnam, Laos, Thailand, Myanmar, and NE India [1]. After it was established by Schlechter [2], *Holcoglossum* remained monotypic for over 50 years until Garay [3] delimited the genus and transferred *Vanda kimballiana* Rchb. f. and *Vanda rupestris* Hand.-Mazz. to it. In 1982, Tsi [4] added two species, *Holcoglossum flavescens* (Schltr.) Z. H. Tsi and *H. junceum* Tsi, though the latter was reduced to a synonym of *Ascocentrum himalaicum* (Deb, Sengupta & Malick) Christenson by Christenson in 1987 [5]. After an examination of the “Vanda-Aerides alliance”, Christenson [5] assigned *Vanda subulifolia* Rchb. f. and *Vanda amesiana* Rchb. f. to *Holcoglossum*, and further divided *Holcoglossum* into two sections, *H.* sect. *Kimballianum* and *H.* sect. *Holcoglossum*, based on plant habit and flower number per inflorescence. Seidenfaden [6], [7] disagreed with Christenson's treatment, however, and suggested that a new genus is necessary for *Vanda amesiana* and *V. subulifolia.* For the latter 2 species, Jin [8] proposed a new subgenus of *Holcoglossum*, *H.* subgen. *Brachycentron.* This subgenus is characterized by its lip shallowly saccate at the base, with a ridged callus on the front margin of the sac mouth, and an oblong or linear stipe with its base as broad as its apex. In the typical subgenus *H.* subgen. *Holcoglossum*, the lip has a conspicuous spur at the base and a crested or fleshy callus on the mid-lobe. The stipe is tapered, with one end broader than the other. Jin [8] further divided the latter subgenus into two sections: *H.* sect. *Holcoglossum* and *H.* sect. *Sorotylos*. Based on nuclear ITS and chloroplast *trnL-F* and *matK* analyses, Fan et. al. [9] suggested that *Holcoglossum* is a monophyletic genus consisting of three major subclades, albeit including two debatable species, *H. amesianum* and *H. subulifolium*. They even treated *H. kimballianum* and *H. wangii* as being closely related to the two debatable species, and grouped them together into the same clade (tropical clade). Morphologically, however, *H. kimballianum* and *H. wangii* are quite different from *H. amesianum* and *H. subulifolium*, and they were once regarded as belonging to two different sections [5], [10] or even two different subgenera [8]. In fact, according to the molecular evidence provided by Fan et al. [9], the systematic positions of *H. kimballianum* and *H. wangii* are still not clear. This treatment is problematic, however, and clarification requires further molecular analysis or repeated tests for further generic delimitation and infrageneric classification.

In this study, phylogenetic relationships between *Holcoglossum*, allied genera, and infrageneric taxa were assessed using longer gene sequences for nuclear ITS, chloroplast *trnL-F*, and *matK* than employed previously [9], in combination with morphological analyses.

## Results

The DNA sequences of 31 taxa were obtained and analyzed. The DNA sequences of 15 species (14 species of *Holcoglossum* and 1 species of *Papilionanthe*) were newly obtained except for the *matK* fragments from three species. Most of the sequences we obtained are longer than those used in previous research [9]. Detailed sequence information is listed in supplementary Table S2, and can also be accessed from GenBank. The aligned length, the indels' information, the numbers of variable sites and parsimony informative sites, tree statistics for the maximum parsimony (MP) analysis, and the best-fit model selected by Modeltest are given in Tables 1 and 2. Genetic distances between species and between sections of *Holcoglossum* are given in Table 3.

**Table 1:** Statistics from the analyses of the various datasets.

Information	ITS	*trnL-F*	*matK*	Combined
No. of taxa	31	31	31	31
Aligned length	688	1590	1757	4035
No. variable characters	196	337	411	944
No. informative characters(%)	114(16.6)	159(10.0)	156(8.9)	429(10.6)
Tree length	347	442	552	1455
Consistency index	0.6801	0.8348	0.8333	0.7331
Retention index	0.7511	0.8685	0.7870	0.7216
Rescaled consistency index	0.5108	0.7250	0.6559	0.5290
Indels	8	36	8	52

**Table 2:** Best-fit model and parameter for each dataset.

Region	AIC select model	Base frequencies				substitution model(rate matrix)						I	G
		A	C	G	T	A–C	A–G	A–T	C–G	C–T	G–T		
ITS	TrN+I+G	0.1951	0.3045	0.3301	0.1704	1.0000	4.1184	1.0000	1.0000	6.2680	1.0000	0.4216	0.9399
*trnL-F*	TVM+I+G	0.3661	0.1329	0.1225	0.3785	0.8927	0.9529	0.7290	0.1252	0.9529	1.0000	0.3369	0.7421
*matK*	K81uf+I+G	0.3110	0.1651	0.1471	0.3769	1.0000	1.2207	0.4074	0.4074	1.2207	1.0000	0.3290	0.8743
Combined	K81uf+I+G	0.3098	0.1776	0.1680	0.3446	1.0000	1.6269	0.6450	0.6450	1.6269	1.0000	0.4392	0.7241

**Table 3:** Interspecific genetic distances of *Holcoglossum* clade based on combined datasets of ITS, *trnL-F*, and *matK*.

		1	2	3	4	5	6	7	8	9	10
1	*H. rupestre*	-									
2	*H. flavescens*	0.0050	-								
3	*H. sinicum*	0.0050	0.0067	-							
4	*H. weixiense*	0.0047	0.0063	0.0037	-						
5	*H. quasipinifolium*	0.0151	0.0179	0.0124	0.0148	-					
6	*H. tsii*	0.0053	0.0084	0.0070	0.0060	0.0131	-				
7	*H. lingulatum*	0.0097	0.0128	0.0080	0.0097	0.0141	0.0063	-			
8	*H. omeiense*	0.0097	0.0128	0.0080	0.0097	0.0141	0.0063	0.0000	-		
9	*H. linearifolium*	0.0237	0.0261	0.0240	0.0237	0.0313	0.0220	0.0278	0.0278	-	
10	*H. nujiangense*	0.0158	0.0175	0.0155	0.0151	0.0257	0.0155	0.0213	0.0213	0.0090	-

### ITS analysis

This analysis strongly supports *Holcoglossum auriculatum* as a sister to *Papilionanthe teres* and *P. biswasiana*, the posterior probabilities (PP) is 100%. *Holcoglossum* can be divided into three major clades. The first clade includes *H. amesianum*, *H. subulifolium*, *H. kimballianum*, and *H. wangii* and is a sister to the genus *Papilionanthe* (PP100%). Clade one consists of two groups, one including *H. amesianum* and *H. subulifolium*, and the other *H. wangii* and *H. kimballianum*. Division of the other species into the other two clades are well supported by Bayesian inference (BI) analysis (PP100%), with one clade including *H. flavescens*, *H. rupestre*, *H. tsii*, *H. linearifolium* (new species), and *H. nujiangense*, and the other including *H. weixiense*, *H. quasipinifolium*, *H. omeiense*, *H. lingulatum*, and *H. sinicum*. The relations of other closely related genera are well resolved except for the genus *Aerides*, and the relative species form several clades (see Supplementary Figs. S1, S2, S3).

### Chloroplast *trnL-F* and *matK* data analyses

The consensus trees of *trnL-F* (see Supplementary Figs. S4, S5, S6) demonstrate that *H. kimballianum* and *H. wangii* are weakly supported as a sister to the genus *Rhynchostylis* (PP77%), and they are not included in the main clade of *Holcoglossum*. The main clade of *Holcoglossum* is divided into four subclades. The first subclade includes *H. amesianum*, *H. subulifolium*, and *H. auriculatum*; the second subclade includes *H. omeiense* and *H. lingulatum*; the third subclade includes *H. tsii*, *H. weixiense*, *H. flavescens*, and *H. rupestre*, and the fourth subclade consists of *H. quasipinifolium*, *H. sinicum*, *H. linearifolium*, and *H. nujiangense*. The BI and maximum likelihood (ML) trees yield the same topological structures, but *H. tsii* in the MP tree is included in the fourth subclade. The relations of other closely related genera are also well resolved except for *Aerides*, and the relative species formed several clades.

The consensus trees of *matK* are less clear, but *H. kimballianum* and *H. wangii* are more closely related to *Vanda* than to any other species of *Holcoglossum*, and *H. linearifolium* and *H. nujiangense* are well supported as sisters to the genus *Papilionanthe*. (see Supplementary Figs. S7, S8, S9).

### Morphological analyses

The morphological character matrix phylogenetic tree of *Holcoglossum* supports the division of *Holcoglossum* into three clades: the first clade consisting of *H. kimballianum* and *H. wangii*, the second consisting of *H. amesianum*, *H. subulifolium* and *H. auriculatum*, and the third including the remaining species of *Holcoglossum*.

### Combined dataset analysis

**Figure 1:**
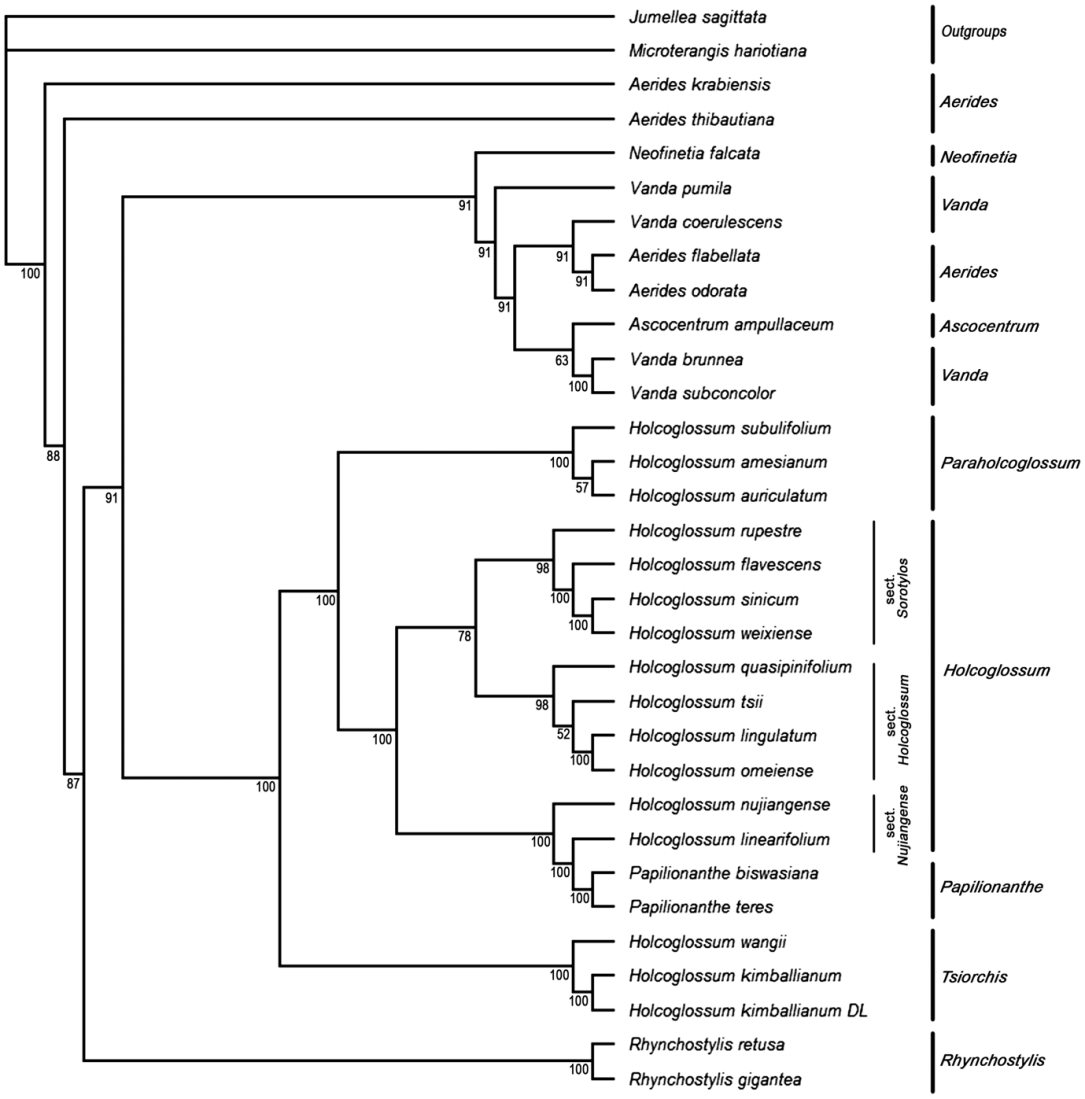
Bayesian consensus trees based on the last 30,001 maximum likelihood trees for the combined datasets of ITS, *trnL-F*, and *matK*. The Bayesian posterior probability (×100) is given below the branches.

In the present study, we also combined ITS, *trnL-F*, and *matK* into a single dataset. The strict consensus trees strongly support the division of *Holcoglossum* into three clades (PP100%). The first clade, consisting of *H. kimballianum* and *H. wangii*, is strongly supported as a sister to the genera *Rhynchostylis* and *Vanda* (PP100%). The second clade, consisting of *H. amesianum*, *H. subulifolium*, and *H. auriculatum*, is strongly supported as a sister to the first clade (PP100%). These two clades are not included in main clade of *Holcoglossum*. The last clade includes the remaining species of *Holcoglossum* (PP100%) and is further divided into three subclades or sections. The first subclade, *Holcoglossum* sect. *Holcoglossum*, consists of four species: *H. tsii*, *H. quasipinifolium*, *H. omeiense*, and *H. lingulatum*. The second subclade, *H.* sect. *Sorotylos*, also contains four species, *H. rupestre*, *H. sinicum*, *H. flavescens*, and *H. weixiense*, while the third, *H.* sect. *Nujiangensia*, consists of *H. nujiangense* and *H. linearifolium*. The latter subclade is well supported as a sister to the genus *Papilionanthe* (Fig. 1; Supplementary Figs. S10, S11).

**Figure 2:**
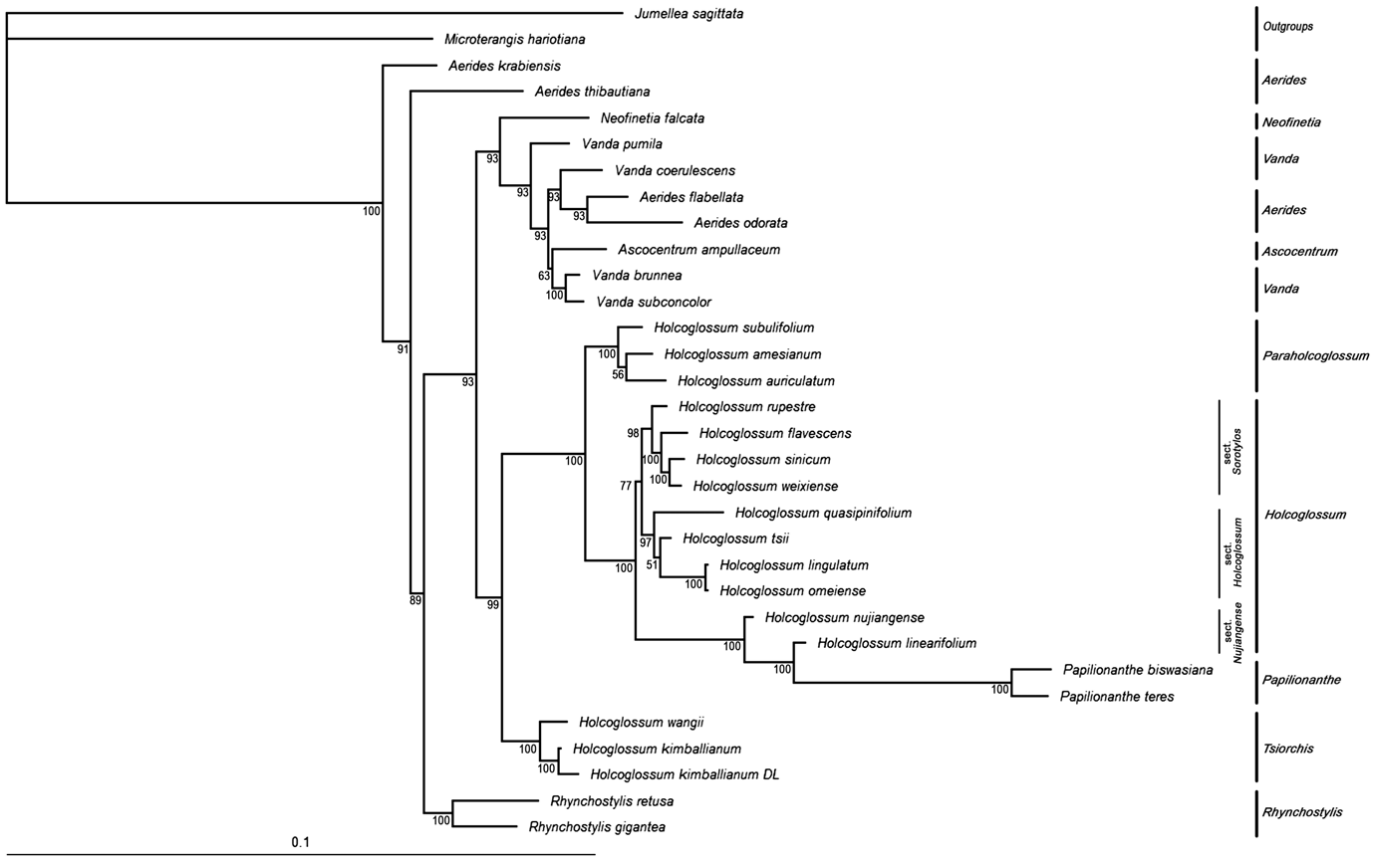
One phylogenetic tree of Bayesian inference consensus trees based on the last 30,001 maximum likelihood trees for the combined datasets of ITS, *trnL-F*, *matK*, and morphological characters matrix. The Bayesian posterior probability (×100) is given near the node.

In the present study, we also combined molecular data and morphological characters in a single dataset. The BI consensus tree of this dataset has similar topology to that constructed from the combined data set from ITS, *trnL-F*, and *matK* analysis, but most of the clades and subclades are better delimited and more strongly supported (Fig. 2).

Among the 14 analyzed species in six genera (Aeridinae), the species of five genera cluster together in the cladogram forming several related clades with the exception of one genus, *Ascocentrum* (*A. ampullaceum*), and two species, *Aerides flabellata* and *Aerides odorata*, which all cluster with *Vanda* forming the *Vanda* clade.

### Genetic distance analyses

In general, the genetic distances (GD) between species within a genus are lower than those between intergeneric species. The mean genetic distance between all the species is 0.0311 when outgroups are included, and the value is 0.0245 when outgroups are excluded. Within *Holcoglossum*, most of the genetic distances between species are lower than 0.0200 (see Supplementary Table S1). The average genetic distance of all the species of *Holcoglossum* is 0.0154. In the *Holcoglossum* clade, the GD values between species are given in Table 3 (the mean genetic distance is 0.0137). The genetic distances between *H. linearifolium* and the other species are generally higher than distances between other species pairs, which strongly supports the treatment of *H. linearifolium* as a new species distinct from all known species.

## Discussion

### Data of ITS, *trnL-F* and *matK* analyses

In the present study, ITS, *trnL-F*, and *matK* were used to resolve the phylogenetic relationships within the genus *Holcoglossum.* We developed five sets of primers to sequence some difficult fragments, but there were still only partial fragments of the *matK* for three species (*H. weixiense*, *H. rupestre*, *H. sinicum*) (see Supplementary Table S2), and the fragments of the *trnL-F* for *H. kimballianum* and *H. subulifolium* have some differences from those reported in a previous study [9]. In the present study, the *trnL-F* of *H. subulifolium* lost 291 bp compared to the sequence described previously [9], so we used the previous sequence. We also used the new sequence in our analysis, however, and results in the combined dataset analysis were the same (see Supplementary Figs. S12, S13, S14). The fragments of *H. kimballianum* in the previous study [9] had one more insert (19 bp) than in our study, so both of them were used for the present analysis. Therefore, these five fragments were accessed from GenBank (see Supplementary Table S2). In total, the fragments we obtained were almost the same as those of Fan et al. [9], but longer (see Supplementary Table S2).

In this study, we found that it was better to utilize ITS and *trnL-F* than *matK* to resolve interspecific relationships within the genus *Holcoglossum*, consistent with the previous study [9]. The ITS and *trnL-F* produced approximately equivalent tree topologies (see Fig. 1 and Supplementary Figs. S1, S2, S3, S4, S4, S6) that agreed with the main clades established by morphological characterization (see Supplementary Fig. S10). The consensus tree of *matK* showed inexact results (see Supplementary Figs. S7, S8, S9), which may be due to insufficient variations in the sequence of *matK*. Many studies had shown that at lower taxonomic levels (tribes and below), non-coding plastid markers (often the *trnL* intron and the *trnL-F* intergenic spacer) and ITS (ITS1-5.8S-ITS2) nuclear ribosomal DNA spacer [11] may yield better results than protein-coding genes such as *rbcL* [12], [13], *matK* [14], *psaB* [15], or *ycf1* [16]. Conversely, at the family level, plastid protein-coding genes have been the primary focus, including as *rbcL* [12], [13], *matK* [14], *psaB* [15], and *ycf1* [16]. Obviously this study focused on lower taxonomic levels (tribes and below), so it was reasonable that the consensus trees of *matK* showed inexact results. When a combined ITS, *trnL-F*, and *matK* dataset was used to analyze these relationships, however, the best tree topology was obtained, and was consistent with the delimitation based on their morphological characters. Using this combined dataset, most of the clades and subclades were better delimited and more strongly supported. From the present study, the *matK* fragments were still useful in helping to resolve the relationships below the generic rank when combined with ITS and *trnL-F*.

In this study, phylogenetic analyses were performed under maximum likelihood (ML), maximum parsimony (MP), and Bayesian inference (BI) for each dataset. The BI, MP, and ML trees of each dataset had similar topological structures, meaning that the analysis had very good repeatability and that the experimental data were reliable. The differences between the BI, MP, and ML trees were the values of bootstrap percentages (BP) or posterior probabilities (PP) in each node; in general, the values in the BI tree were higher than that in MP and ML trees. In this paper, we will use the BI trees to demonstrate phylogenetic relationships between *Holcoglossum* and its allied genera. The MP and ML trees are presented in the Supplementary Information (see Supplementary Figs. S1, S2, S3, S4, S5, S6, S7, S8, S9, S10, S11, S12, S13, S14).

### Phylogeny of *Holcoglossum*

In total, 15 *Holcoglossum* species, including a new one, were analyzed. Analysis of combined chloroplast and nuclear data strongly supported (PP100%) the tripartite division of *Holcoglossum* into the *Holcoglossum* clade, its sister clade, and a distant clade (Fig. 1 and Supplementary Figs. S10, S11, S12, S13, S14). Similar results were obtained by combining molecular data and morphological characters in a single dataset (Fig. 2). On these grounds, the trees of ITS (Supplementary Figs. S1, S2, S3), *trnL-F* (Supplementary Figs. S4, S5, S6) and the combinational dataset produced highly similar tree topologies. The main clades established were supported by these datasets. The phylogenetic relationships between *Holcoglossum* and allied genera were well resolved, and all data sets supported the division of *Holcoglossum* into three clades. Although the consensus trees of *matK* (Supplementary Figs. S7, S8, S9) showed inexact results, it supported the trees of *trnL-F*, showing that *H. wangii* and *H. kimballianum* form a new clade outside the *Holcoglossum* clade. The BI consensus trees of ITS, *trnL-F*, and combinational dataset were similar, but most of the clades and subclades were better delimited and more strongly supported by the combinational dataset, implying that combinational datasets may be better than single genes for phylogenetic analysis.

From the tree of the combinational dataset, the *Holcoglossum* clade was a sister to *Papilionanthe*, a genus of some ten species, while *H. subulifolium*, *H. auriculatum*, *H. amesianum*, *H. kimballianum*, and *H. wangii* were not included in the *Holcoglossum* clade. They formed two clades outside the main *Holcoglossum* clade. In fact, *H. kimballianum* and *H. wangii* were more closely related to *Vanda* than to the main *Holcoglossum* clade in the *trnL-F* and *matK* dataset. Moreover, the clade sequences are considerably different from each other, especially in the *trnL-F* sequence. In order to explain the differences between them, we listed some obvious differences in the *trnL-F* aligned file (see Supplementary Figs. S15, S16, S17, S18 for details). This aligned file revealed that each of the three clades had their own unique characters and could be easily distinguished. In particular, *H. kimballianum* and *H. wangii* are quite distinct from the other species. Moreover, the phylogenetic relationships established between species in the three clades by analysis of morphological characters were strikingly similar to the relationships defined by the combined molecular data (Fig. 2). Therefore, we conclude that some species must be classified into two new genera, *Tsiorchis* and *Paraholcoglossum*, distinct from *Holcoglossum*. This conclusion may also be drawn from Jin [8] and Fan et al. [9], but they treated them only at subgeneric rank. As a result, *Tsiorchis* and *Paraholcoglossum* are proposed to be outside the *Holcoglossum* clade.

*Paraholcoglossum* comprises three species, *Paraholcoglossum subulifolium*, *P. auriculatum*, and *P. amesianum*, while *Tsiorchis* consists of two species, *Tsiorchis kimballiana* and *T. wangii. Holcoglossum* includes the remaining ten species. The three genera show obvious morphological differences. In *Holcoglossum*, the white lip is conspicuously spurred at the base, with a crested or fleshy callus at the broad base of the mid-lobe, and the pollinia are porate and directly attached to a tapering stipe (see Supplementary Fig. S19). In *Paraholcoglossum*, the lip is shallowly saccate at the base, with a ridged callus at the entrance to the sac, and the mid-lobe is clawed at the base. The pollinia is porate in *Paraholcoglossum*, but the stipe is oblong, with both ends almost equal in width (see Supplementary Fig. S20). *Tsiorchis* is quite distinct from *Holcoglossum* and *Paraholcoglossum*, having a cleft, not a porate pollinia; each pollinium has a distinct caudicle attached to a common stipe; the mid-lobe of the lip is purple or has purple markings and the side-lobe is yellow with dark-purpled markings (see Supplementary Fig. S21).

Within the genus *Holcoglossum*, the infrageneric system proposed by Jin [8] and Fan et al. [9] accurately reflect many interspecies relationships, though some changes are made in our system (Fig. 1, 2). We also analyzed the genetic distances between the sections of *Holcoglossum*. The average genetic distances between species in different sections are 0.0052 for *H.* sect. *Holcoglossum*, 0.0090 for *H.* sect. *Sorotylos*, and 0.0090 for *H.* sect. *Nujiangensia.* The genetic distances between sections are 0.0105 (*H.* sect. *Holcoglossum* and *H.* sect. *Sorotylos*), 0.0202 (*H.* sect. *Holcoglossum* and *H.* sect. *Nujiangensia*), and 0.0241 (*H.* sect. *Nujiangensia* and *H.* sect. *Sorotylos*) (see Supplementary Table S3). This result is consistent with topological analysis. The morphological characters and molecular evidence both support the division of *Holcoglossum* into three subclades (Figs. 1, 2). The first subclade, *Holcoglossum* sect. *Holcoglossum*, includes four species, *H. tsii*, *H. quasipinifolium*, *H. omeiense* and *H. lingulatum*, all of which are characterized by their mid-lobe of lip arising from the upper third of the spur, ligulate mid-lobe twice as long as the spur, mid-lobe with 2–7 lamellae adaxially (see Supplementary Fig. S19. a–c). The second subclade, *Holcoglossum* sect. *Nujiangensia*, is a new section with two species, *H. nujiangense* and *H. linearifolium* sp. nov., and is characterized by a mid-lobe of lip arising from the middle of the spur, obovate almost as long as the spur, and 3–5 crested lamellae extending down to the middle of the spur (see Supplementary Fig. S19. d, e). *Holcoglossum* sect. *Nujiangensia* is the immediate sister to *Holcoglossum* sect. *Sorotylos* and *H.* sect. *Holcoglossum*. The third subclade is *Holcoglossum* sect. *Sorotylos*, which includes four species: *H. rupestre*, *H. sinicum*. *H. flavescens*, and *H. weixiense*, all characterized by a lip with a shorter tapering spur and crest-like calli on the mid-lobe (see Supplementary Fig. S19. f, g).

We also assessed the genetic distances between the individual species of main *Holcoglossum* clade. The mean interspecies genetic distance is 0.0137 (Table 3) and pair-wise distances range from 0.0000 (*H. omeiense* and *H. lingulatum*) to 0.0313 (*H. linearifolium* and *H. quasipinifolium*), while the genetic distances between *H. linearifolium* and other species range from 0.0090 to 0.0313, with a mean (0.0215) significantly higher than the average interspecific genetic distance within the genus *Holcoglossum*. The genetic distance between *H. linearifolium* and *H. nujiangense* is 0.0090, which is also much higher than that between most other species pairs, such as *H. weixiense* and *H. sinicum* (0.0037), *H. tsii* and *H. rupestre* (0.0053), or *H. rupestre* and *H. weixiense* (0.0047) (Table 3). Considering these molecular results as well as morphological characters, *H. linearifolium* is proposed as a new species that differs from its ally *H. nujiangense* by having a stem 4–5 cm long, filiform, soft leaves about 1 mm thick and often 30–40 cm long, side-lobes of lip narrowly ovate (not triangular), and mid-lobe obovate 8–10 mm in length.

Among the 14 analyzed species in six genera (Aeridinae), the species of five genera cluster together in the cladograms forming several related clades. However *Aerides flabellata*, *Aerides odorata*, and *Ascocentrum ampullaceum* cluster together with *Vanda* to form the *Vanda* clade, probably due to some species missing partial fragments of *trnL-F* or *matK* region. (see Supplementary Table S2). *Papilionanthe* teres and *P. biswasiana* cluster together with the *Holcoglossum* sect. *Nujiangensia*. The results indicate that analysis of longer DNA sequences is helpful for resolving the relationships below generic rank.

### Conclusion

In the present study, we investigated the phylogenetic relationships between *Holcoglossum* and allied genera, as well as the infrageneric classification of the genus *Holcoglossum* based on both morphological characters and molecular evidence. *Holcoglossum* is triphyletic and can be divided into two major sister clades and a distant clade. The three clades are regarded as three genera: *Holcoglossum*, *Paraholcoglossum*, and *Tsiorchis*. The latter two are published here as new genera. *Paraholcoglossum* includes three species and is related to *Holcoglossum*, but is quite distinct in many respects. *Tsiorchis* includes two species and is more closely related to *Vanda* than to *Holcoglossum*. The genus *Holcoglossum* itself comprises three subclades, all recognized here at sectional rank: *H.* sect. *Holcoglossum* with four species, *H.* sect. *Sorotylos* with four species and *H.* sect. *Nujiangensia* with two species (Fig. 1, 2).

Taxonomic treatment

**Figure 3:**
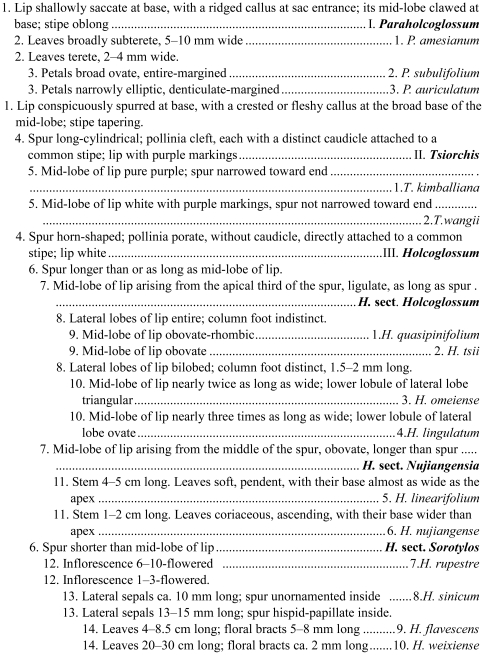
Key to the genera, sections, and species of *Paraholcoglossum*, *Tsiorchis*, and *Holcoglossum*.

Key to the genera, sections, and species of *Paraholcoglossum*, *Tsiorchis*, and *Holcoglossum* shown in Fig. 3.

**I.** ***Paraholcoglossum*** Z. J. Liu, S. C. Chen & L. J. Chen, gen. nov. [urn:lsid:ipni.org:names: 77113675–1]

· **Diagnosis**. Genus novum *Holcoglosso* affine, a quo labello saccato prope basin, callo porcato ad marginem anticum oris sacci praedito, lobo intermedio unguiculato ad basin, stipite oblongo, ejus basi et apice aequilato.

The new genus is akin to *Holcoglossum*, from which it differs by its lip saccate at base, with a ridged callus on the front margin of the sac mouth, mid-lobe clawed at base, and stipe oblong, with its base as wide as apex.

· **Description**. Epiphytic plants. Stems often elongate. Leaves broadly subterete to terete, adaxially channeled. Inflorescence racemose or paniculate (with 1–2 branches), with several to many flowers; bracts much shorter than pedicellate ovary; flowers open widely; sepals and petals subsimilar; lateral sepals usually slightly larger; lip saccate at base, trilobed, with a ridged callus at the front margin of the sac mouth; mid-lobe clawed at base; column short, winged, with a very short but distinct foot; pollinia 2, waxy, globose, porate, attached by an oblong stipe to a broad viscidium.

· **Type:** ***Paraholcoglossum amesianum*** (Rchb. f.) Z. J. Liu, S. C. Chen & L. J. Chen

1. ***Paraholcoglossum amesianum*** (Rchb. f.) Z. J. Liu, S. C. Chen & L. J. Chen, comb. nov. [urn:lsid:ipni.org:names: 77113676–1]

· **Type**: Myanmar, Shan State, Herb. Reichenbach 37196 (holotype, W).

*Vanda amesiana* Rchb. f. in Gard. Chron., ser. 3, 1: 764. 1887.

*Holcoglossum amesianum* (Rchb. f.) Christenson in Notes Roy. Bot. Gard. Edinburgh 44: 255. 1987.

· **Distribution**: China, India, Laos, Myanmar, Thailand, Vietnam.

· **Habitat**: On tree trunks; 1200–2000 m.

2. ***Paraholcoglossum subulifolium*** (Rchb. f.) Z. J. Liu, S. C. Chen & L. J. Chen, comb. nov. [urn:lsid:ipni.org:names: 77113677–1]

· **Type**: Myanmar, Herb. Reichenbach 37215 (holotype, W).

*Vanda subulifolia* Rchb. f., Flora 69: 552. 1886.

*Vanda watsonii* Rolfe in Gard. Chron. Ser. 3, 37: 82, 123. 1906.

*Holcoglossum subulifolium* (Rchb. f.) Christenson in Notes Roy. Bot. Gard. Edinburgh 44: 255. 1987.

· **Distribution**: China, Myanmar, Thailand, Vietnam.

· **Habitat**: On tree trunks; 1300–2200 m.

3. ***Paraholcoglossum auriculatum*** (Z. J. Liu, S. C. Chen & X. H. Jin) Z. J. Liu, S. C. Chen & L. J. Chen, comb. nov. [urn:lsid:ipni.org:names: 77113678–1]

· **Type**: China, NE Yunnan, Malipo, 16 May 2003, Z. J. Liu 2758 (holotype, NOCC!)

*Holcoglossum auriculatum* Z. J. Liu, S. C. Chen & X. H. Jin in *J. Wuhan Bot. Res.,* 23 (2):154–156. 2005.

· **Distribution**: China, NE Yunnan (Malipo County).

· **Habitat**: On tree trunks; 2200 m.

· **Note**: This is a distinct species based on both molecular and morphological data.

**II.** ***Tsiorchis*** Z. J. Liu, S. C. Chen & L. J. Chen, gen. nov. [urn:lsid:ipni.org:names: 77113679–1]

· **Diagnosis**. Genus novum *Holcoglosso*, *Rhynchostyli* et *Vandae* subsimile, differt a *Holcoglosso* polliniis fissis (non poratis) caudicula una distincta in quoque pollinio, labello purpureo-marmorato, calcari cylindrico, a *Rhynchostyle* foliis teretibus, labello trilobo lobis lateralibus conspicuis praedito, columna sine pede, stipite breviore latioreque, a *Vanda* foliis teretibus, caudicula praesenti, calcari cylindrico.

The new genus is subsimilar to *Holcoglossum*, *Rhynchostylis*, and *Vanda*. It differs from *Holcoglossum* by its cleft (not porate) pollinia, each with a distinct caudicle, purple-mottled lip and cylindrical spur; from *Rhynchostylis* by its terete leaves, trilobed lip with conspicuous side-lobes at base, footless column and shorter and broader stipe; from *Vanda* by its terete leaves, distinct caudicle and cylindrical spur.

· **Description**. Epiphytic plants. Stems often elongate. Leaves terete, adaxially channeled. Inflorescence racemose, often nodding, with many flowers; bracts small; flowers opening widely; dorsal sepal and petals subsimilar; lateral sepals slightly larger; lip with purple markings, trilobed, conspicuously spurred at base; spur long-cylindrical, mid-lobe suborbicular, not clawed at base; column footless; pollinia waxy, subglobose, cleft, each with a distinct caudicle attached by a common tapering stipe to a broad viscidium.

· **Type:** ***Tsiorchis kimballiana*** (Rchb. f.) Z. J. Liu, S. C. Chen & L. J. Chen

1. ***Tsiorchis kimballiana*** (Rchb. f.) Z. J. Liu, S. C. Chen & L. J. Chen, comb. nov. [urn:lsid:ipni.org:names: 77113680–1]

· **Type**: Myanmar, southern Shan States, Herb. Reichenbach 37216 (holotype, W).

*Vanda kimballiana* Rchb. f. in Gard. Chron, ser. 3, 5: 232. 1889.

*Vanda saprophytica* Gagnep. in Bull. Soc. Bot. Fr. 79:37, 1932.

*Holcoglossum saprophyticum* (Gagnep.) Christenson in Not. Bot. Gard. Edinb. 44(2): 255. 1987.

· **Distribution**: China (S Yunnan), Laos, Myanmar, Thailand, NW Vietnam.

**2.** ***Tsiorchis wangii*** (Christenson) Z. J. Liu, S. C. Chen & L. J. Chen, comb. nov. [urn:lsid:ipni.org:names: 77113681–1]

· **Type**: China, Yunnan, Hort. Mountain. Orchids s.n. (holotype, K in spirit).

*Holcoglossum wangii* Christenson in Lindleyana 13: 123. 1998.

· **Distribution**: China, SW Guangxi and SE Yunnan.

· **Habitat**: On tree trunks in broad-leaved evergreen forests; 800–1200 m.

**III.** ***Holcoglossum*** Schlechter in Repert, Spec. Nov. Regni Veg. Beih. 4: 285. 1919

· **Description**. Epiphytic plants. Stems short. Leaves many, distichous, terete, subterete, or broadly subterete, fleshy, adaxially channeled, jointed and dilated into sheathing base. Inflorescences axillary, racemose, with few to many flowers; rachis usually purple; flowers usually opening widely; sepals and petals subsimilar, usually ± carinate abaxially; lateral sepals often slightly larger, oblique; lip spurred, 3-lobed; lateral lobes erect beside entrance of spur, spotted; mid-lobe rather larger, often narrowed and with appendages at base; spur often cylindric and curved, attenuated toward tip, usually hispid-papillose inside. Column short, thick, winged, with a very short but distinct foot; pollinia 2, waxy, globose, porate, attached by a common, linear stipe to a broad viscidium.

· **Type:** ***Holcoglossum quasipinifolium*** (Hayata) Schlechter.

*Holcoglossum* sect. *Holcoglossum*

1. ***Holcoglossum quasipinifolium*** (Hayata) Schltr. In Fedde Repert. Sp. Nov. Beih. 4: 285. 1919.

· **Type**: China, Taiwan, Mt. Arisan, Hayata & Sasaki s.n. (holotype, T).

*Saccolabium quasipinifolium* Hayata, Icon. Pl. Formos, 2: 144. 1912.

· **Distribution**: China, C Taiwan, Jiayi county and Xinju county.

· **Habitat**: On tree trunks in mixed or coniferous forests; 1800–2800 m.

2. ***Holcoglossum tsii*** T. Yukawa in Ann. Tsukuba Bot. Gard. 19: 1. 2000.

· **Type**: China, Yunnan, TNS 9512285 (holotype, Hort. Tsukuba Bot. Gard; isotype PE!)

· **Distribution**: China, Yunnan, no precise locality.

· **Habitat**: Unknown.

3. ***Holcoglossum omeiense*** X. H. Jin et S. C. Chen in Kew Bull. 59: 633. 2005.

· **Type**: China, Sichuan, Mt. Emei, K. H. Shing et K. Y. Lang 1365A (holotype, PE!)

· **Distribution**: Known only from type locality.

· **Habitat**: On tree trunks in open forest; 700–1000 m.

4. ***Holcoglossum lingulatum*** (Averyanov) Averyanov, Konsp. Sosud. Rast. Fl. Vetnama 1: 110. 1990.

· **Type**: Vietnam, between Chapa and Hoan Lien Song, Takhtajan 0745 (holotype, LE).

*Holcoglossum kimballianum* (Rchb. f.) Garay var. *lingulatum* Averyanov in Bot. Zhurn. (Mosow & Leningrad) 73: 426. 1988.

*Holcoglossum tangii* Christenson in Lindleyana 13 (2): 121–124. 1999. syn. nov.

· **Distribution**: China (SE Yunnan), Vietnam.

· **Habitat**: On tree trunks in open forests; 1000–1400 m.

*Holcoglossum* sect. *Nujiangensia* Z. J. Liu, S. C. Chen & L. J. Chen sect. nov. [urn:lsid:ipni.org:names: 77113682–1]

· **Diagnosis**. Sectio nova *Holcoglosso* sect. *Holcoglosso* similis, a quo differt lobo intermedio labelli ad medium calcaris exorienti obovato calcari longiore.

New section is similar to *Holcoglossum* sect. *Holcoglossum*, from which it differs by the mid-lobe of lip arising from the middle of the spur, obovate, longer than spur.

· **Type**: ***Holcoglossum nujiangense*** X. H. Jin & H. Li

**Figure 4:**
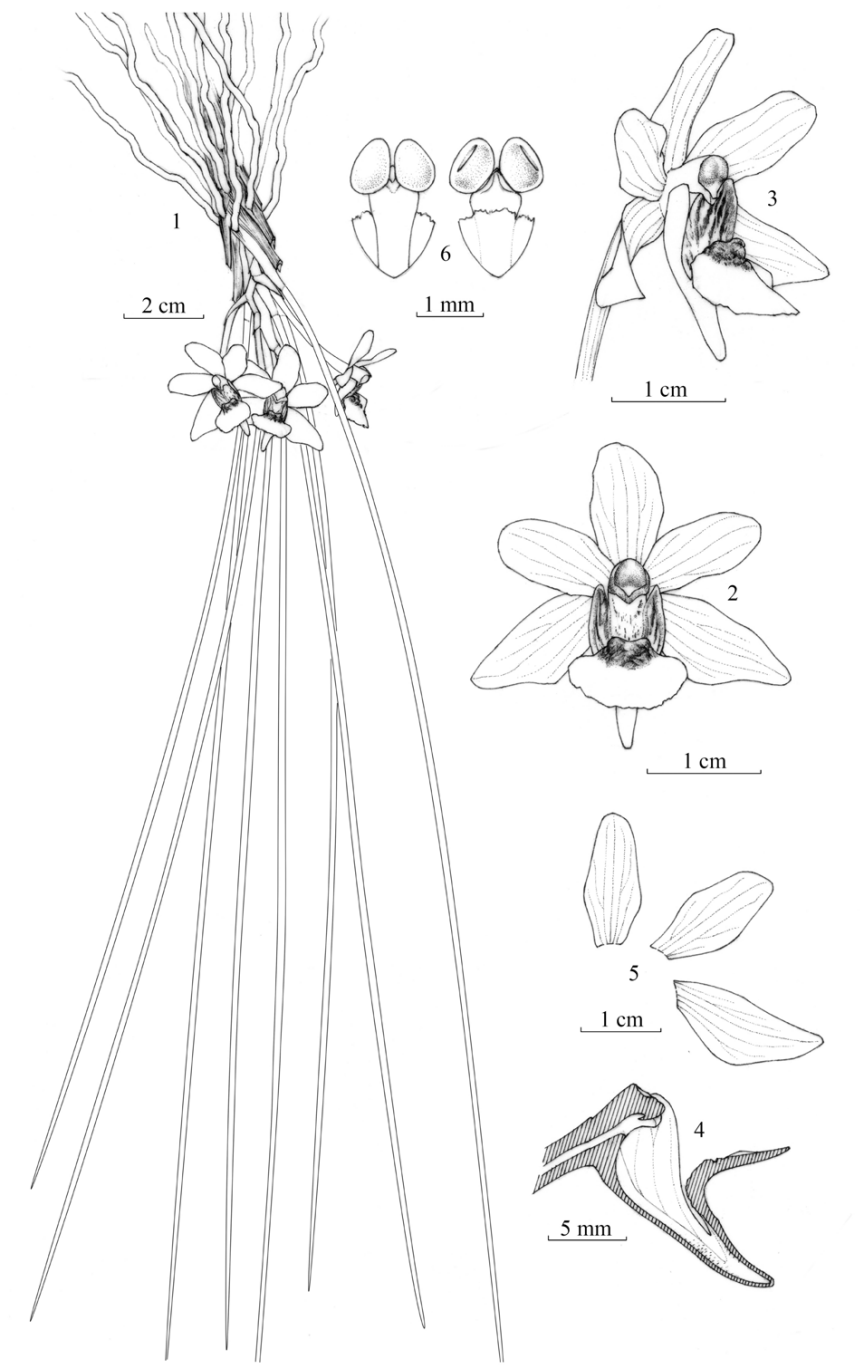
*Holcoglossum linearifolium* Z. J. Liu, S. C. Chen & L. J. Chen: **1.** Flowering plant; 2. Flower, front view; 3. Flower, side view; 4. Lip and column, longitudinal section; 5. Dorsal sepal, petal and lateral sepal; 6. Pollinarium, front view and back view (Drawn by X. Y. Ma from type Z. J. Liu 4865).

5. ***Holcoglossum linearifolium*** Z. J. Liu, S. C. Chen & L. J. Chen, sp. nov. [urn:lsid:ipni.org:names: 77113683–1] (Fig. 4 & Supplementary Fig. S22).

· **Type:** China, Yunnan, Malipo, alt. 1600 m, 27 March 2010, Z. J. Liu 4865 (holotype, NOCC!); China, Yunnan, Malipo, alt. 1500, 28 March 2010, Z. J. Liu 4948 (NOCC!); China, Yunnan, Malipo, alt. 2000, 28 March 2010, Z. J. Liu 4950 (NOCC!).

· **Diagnosis**. Species nova *Holcoglosso nujiangensi* similis, a quo differt caule 4–5 cm longo, foliis filiformibus mollibus ca. 1 mm in diam. saepe 30–40 cm longis, lobis lateralibus labelli anguste ovatis (non triangu), ejus lobo intermedio obovato 8–10 mm longo.

New species is similar to *Holcoglossum nujiangense*, from which it differs by having a stem 4–5 cm long, filiform, soft leaves ca. 1 mm thick and often 30–40 cm long, side-lobes of lip narrowly ovate (not triangular), and mid-lobe obovate, 8–10 mm long.

· **Description**. Epiphytic plants. Stem 4–5 cm long. Leaves 6–9, filiform, soft, pendent, with their base almost as wide as the apex, inconspicuously channeled adaxially, 30–40 cm long, ca. 1 mm thick, acuminate at apex, jointed and dilated into sheathing base. Raceme 1–2 cm long, with 2–3 flowers; floral bracts ovate, 5–7 mm long; flowers white; sepals and petals with purple-red midrib abaxially; lip with purple-red stripes on side-lobes; dorsal sepal elliptic, 10–11×4.5–5 mm, obtuse at apex; lateral sepals obliquely ovate-elliptic, 11–12.5×5.5–6 mm, subobtuse at apex; petals elliptic, 9.5–10.5×4.5–4.8 mm, rounded at apex; lip adnate to column foot, 3-lobed; side-lobes erect, narrowly ovate, 6–7×2.5–3 mm, acuminate at apex; mid-lobe arising from the middle of the spur, obovate, 8–10 mm long, rounded at apex, base with a very thick callus ridged-swollen on both sides; spur narrowly conical, 5–6 mm long, acuminate at the end; column 3–3.5 mm long, with a foot 1–1.5 mm long. Pollinia 2; stipe tapering. Flowering period: March and April.

· **Distribution**: China, SE Yunnan (Malipo County).

· **Habitat**: Epiphytic, pendulous, on tree trunks in broad-leaved forests, 1500–2000 m.

6. ***Holcoglossum nujiangense*** X. H. Jin & H. Li in Nordic J. Bot. 25: 127. 2008.

· **Type**: China, Yunnan. Fugong, Jiakeding, 2400 m, 16 May 2005, X. H. Jin 6930 (PE).

· **Distribution**: China, W Yunnan.

· **Habitat**: On tree trunks in broad-leaved evergreen forests; 2500–3000 m.

*Holcoglossum* sect. *Sorotylos* X. H. Jin & S. C. Chen

7. ***Holcoglossum rupestre*** (Hand.-Mzt.) Garay in Bot. Mus. Leafl. 23 (4): 182. 1972.

· **Type**: China, Yunnan, Zhongdian (now Sangrilla), Handel-Mazzett 8802 (holotype, W; isotypes Wu, E, K).

*Vanda rupestris* Hand.-Mzt. in Anz. Akad. Wiss. Wien, Math.-Naturwiss. Kl. 62: 241. 1925.

· **Distribution**: NW Yunnan, Sangrilla.

· **Habitat**: On Quercus trunks and branches in mixed forests; 2000–2400 m.

8. ***Holcoglossum sinicum*** Christenson in Notes. Roy. Bot. Gard. Edinburgh 44: 255. 1987.

· **Type**: China, Yunnan, Yanbi, SEBC 380 (holotype, E; isotypes Kun, AMES).

· **Distribution**: China, N Yunnan.

· **Habitat**: On tree trunks in *Alnus* or *Quercus* forests; 2600–3200 m.

9. ***Holcoglossum flavescens*** (Schltr.) Z. H. Tsi in Acta Phytotax. Sin. 20: 441. 1982.

· **Type**: China, Yunnan, Yunpe, Simeon Ten 23 (holotype, BD).

*Aerides flavescens* Schltr. in Fedde Rep. Sp. Nov. 19: 382. 1924.

*Saccolabium yunpeense* T. Tang & F. T. Wang in Acta Phytotax. 1: 97. 1951.

*Papilionanthe flavescens* (Schltr.) Garay in Bot. Mus. Leafl. Harvard Univ. 23 (4): 270. 1972.

· **Distribution**: China, N Fujian, SW Hubei, SW Sichuan and N Yunnan.

· **Habitat**: On tree trunks in broad-leaved evergreen forests; 1200–2700 m.

10. ***Holcoglossum weixiense*** X. H. Jin & S. C. Chen, Syst. Gen. Holcoglossum: 94. 2003.

· **Type**: China, Yunnan, Weixi, HK Kadoorie PT 3490 (holotype, PE).

· **Distribution**: China, NW Yunnan.

· **Habitat**: On tree trunks in broad-leaved forests along valleys; 2500–3000 m.

## Materials and Methods

### Materials

In total, 15 species of *Holcoglossum* were analyzed. Fourteen of them were sampled in this study, while one, *H. tsii*, was accessed from the GenBank. Fourteen additional species, representing six closely related genera, *Aerides*, *Ascocentrum*, *Neofinetia*, *Papilionanthe*, *Rhynchostylis*, and *Vanda*, were accessed from the GenBank and were treated as an ingroup in order to test the monophyly of *Holcoglossum* and to interpret its genetic relationships. Two African orchids of Vadeae, *Jumellea sagittata*, and *Microterangis hariotiana*, were chosen as outgroups [17], [18]. For information regarding the assessment, see Supplementary Table S2 for details.

### Amplification and sequencing

Total DNA was extracted from fresh material or silica-gel-dried plant tissue with a Multisource Genomic DNA Miniprep Kit (Axygen Biosciences) following the manufacturer's instructions. The amplification reaction included total DNA, primers, Ex-Taq buffer, and Ex-Taq DNA polymerase (Takara Bio). The polymerase chain reaction (PCR) profile consisted of an initial 5 min pre-melt stage at 95°C, then 30 cycles of 30 s at 95°C (denaturation), 30 s at 50–55°C (annealing temperature was determined by primer's need), and 1–3 min at 72°C (extension time was determined by length of the aim DNA region), followed by a final 10 min extension at 72°C.

The amplification of the ITS region was performed using the primer pairs ITS A and ITS B [19]. The *trnL-F* region was amplified with primers c and f [20] or two sets of primers developed specifically for this study (Table 4). For *matK* sequences, amplification was performed using the primer pair *matK*-*19F* and *trnK*-*2R* [19], and some fragments were amplified using three sets of primers developed for this study (Table 4). To check the quality of the amplified DNA, PCR products were run on 1.5% agarose gels. The gels with target products were excised, purified using DNA Gel Extraction Kits (Axygen Biosciences), and sequenced by BGI Americas Corporation.

**Table 4:** Primers used in this study.

Primer	Sequence (5′→3′)	Origin
ITS A	GGAAGGAGAAGTCGTAACAAGG	other
ITS B	CTTTTCCTCCGCTTATTGATATG	other
*trnL-C*	CGAAATCGGTAGACGCTACG	other
*trnL-F*	ATITGAACTGGTGACACGAG	other
*trnL-C-70F*	CAAATTCAGAGAAACCCTGGA	this study
*trnL-F-60R*	CCATTTCCCGTGCATCATCCTA	this study
*trnL-MF*	TAAAGAGAGAGTCCCATTTTAC	this study
*trnL-MR*	GAGCGAGGAAGTAAAATGGGC	this study
*trnK-2R*	AACTAGTCGGATGGAGTAG	other
*matK-19F*	CGTTCTCATATTGCACTATG	other
*matK-1867R*	TTGCAGTTTTCATTGCACACG	this study
*matK-147F*	AACAAAACTTCCTATATCCGCT	this study
*matK-1167R*	CATTTGATTTCTTACTACC	this study
*matK-1149F*	GGTAGTAAGAAATCAAATG	this study
*matK-969R*	CTTTTCCTTGATATCGAACAT	this study
*matK-731F*	AAGAAAAGATTCTTTTGGTTCC	this study

### Sequences editing and assembling

Both forward and reverse sequences and electropherograms were edited and assembled with DNASTAR (http://www.dnastar.com/). DNA sequences were aligned with MEGA5.05 [21] under the Muscle model and then adjusted manually with MEGA5.05 [21]. Aligned sequences are available from the corresponding authors upon request.

### Morphological analyses

To explore the phylogenetic positions of the *Holcoglossum* alliance by morphological classification, we constructed a morphological character matrix consisting of 41 characters of 31 taxa (see Supplementary Morphological Character Codes S1and Table S4 for details).

### Data analyses

The datasets included ITS, *matK*, *trnL-F*, and morphological characters, a combination of ITS, *matK* and *trnL-F*, and a combination of all four. Insertions, deletions, and some unavailable sequences were treated as missing. Phylogenetic analyses were performed under maximum likelihood (ML), maximum parsimony (MP), and Bayesian inference (BI). The best fit model for each dataset was selected by Modeltest 3.7 [22] under the Akaike Information Criterion (AIC)(see the Table 3 for the detail).

The MP analyses were performed using PAUP* version 4.0b10 [23]. All characters were equally weighted and unordered. Test settings included 1,000 replications of random addition sequence and heuristic search with tree bisection-reconnection (TBR) branch swapping. The length of tree, consistency indices (CI), and retention indices (RI) are given in Table 2. The ML analysis was computed by RAxML version 7.2.8 with 100 bootstrap replicates and settings as described in Stamatakis et al. [24]. BI analysis was performed using MrBayes 3.1.2 [25]. The best-fit model for each dataset was selected by Modeltest 3.7. In the combined dataset of ITS, *matK* and *trnL-F*, the model was also according to the best fit model for each individual dataset. The following settings were applied: sampling frequency = 100; temp = 0.1; burn-in = 10,000; and the number of Markov chain Monte Carlo (MCMC) generations = 4,000,000. The first 10,000 trees were discarded as burn-in to ensure that the chains reached stationarity. Majority-rule consensus tree was constructed on those trees sampled after generation 1,000,000. We also utilized MEGA5.05 [21] to estimate genetic distances between the species and sections of *Holcoglossum* treated under the Tajima-Nei model based on the combined molecular dataset.

### Nomenclature

The electronic version of this document in itself does not represent a published work according to the International Code of Botanical Nomenclature [26], and hence the new names contained in the electronic version are not effectively published under that Code from the electronic edition alone. Therefore, a separate edition of this document was produced by a method that assures numerous identical printed copies, and those copies were simultaneously distributed (on the publication date noted on the first page of this article) for the purpose of providing a public and permanent scientific record, in accordance with Article 29 of the Code. Copies of the print-only edition of this article were distributed on the publication date to botanical or generally accessible libraries of the following institutions (BM, COL, GH, HUA, K, MEXU, MO, NY, QCA, QCNE, USM). The separate print-only edition is available on request from PLoS (Public Library of Science) by sending a request to PLoS ONE, Public Library of Science, 1160 Battery Street, Suite 100, San Francisco, CA 94111, USA along with a check for $10 (to cover printing and postage) payable to “Public Library of Science”. In addition, new names contained in this work have been submitted to IPNI, from where they will be made available to the Global Names Index. The IPNI LSIDs (Life Science Identifiers) can be resolved and the associated information viewed through any standard web browser by appending the LSID contained in this publication to the prefix http://ipni.org/.

The online version of this work is archived and available from the following digital repositories: PubMedCentral (www.pubmedcentral.nih.gov/).

## Supporting Information

Figure S1 **Bayesian consensus trees based on the last 30,001 maximum likelihood trees for ITS.** The Bayesian posterior probability (×100) is given below the branches.
(TIF)

Figure S2 **The maximum likelihood (ML) trees of ITS computed by RAxML with 100 bootstrap replicates.** The bootstrap values are given below the branches.
(TIF)

Figure S3 **Strict consensus tree of most parsimonious trees based on of ITS sequence data.** Tree length = 347 steps, CI = 0.6801, RI = 0.7511. The bootstrap values of the maximum parsimony analysis are given below the branches.
(TIF)

Figure S4 **Bayesian consensus trees based on the last 30,001 maximum likelihood trees for** ***trnL-F*** **.** The Bayesian posterior probability (×100) is given below the branches.
(TIF)

Figure S5 **The maximum likelihood (ML) trees of** ***trnL-F*** **, computed by RAxML with 100 bootstrap replicates.** The bootstrap values are given below the branches.
(TIF)

Figure S6 **Strict consensus tree of most parsimonious trees based on of** ***trnL-F*** **sequence data.** Tree length = 442 steps, CI = 0.8348, RI = 0.8685. The bootstrap values of the maximum parsimony analysis are given below the branches.
(TIF)

Figure S7 **Bayesian consensus trees based on the last 30,001 maximum likelihood trees for** ***matK*** **.** The Bayesian posterior probability (×100) is given below the branches.
(TIF)

Figure S8 **The maximum likelihood (ML) trees of** ***matK*** **, computed by RAxML with 100 bootstrap replicates.** The bootstrap values are given below the branches.
(TIF)

Figure S9 **Strict consensus tree of most parsimonious trees based on** ***matK*** **sequence data.** Tree length = 552 steps, CI = 0.8333, RI = 0.7870. The bootstrap values of the maximum parsimony analysis are given below the branches.
(TIF)

Figure S10 **The maximum likelihood (ML) trees of ITS,** ***trnL-F*** **and** ***matK*** **, computed by RAxML with 100 bootstrap replicates.** The bootstrap values of are given below the branches.
(TIF)

Figure S11 **Strict consensus tree of most parsimonious trees based on the combined datasets of ITS,** ***trnL-F*** **and** ***matK*** **.** Tree length = 1455 steps, CI = 0.7331, RI = 0.7216. The bootstrap values of the maximum parsimony analysis are given below the branches.
(TIF)

Figure S12 **Bayesian consensus trees based on the last 30,001 maximum likelihood trees for ITS,** ***trnL-F*** **and** ***matK*** **,** ***H. subulifolium*** **re-sequences in this study was included.** The Bayesian posterior probability (×100) is given below the branches.
(TIF)

Figure S13 **The maximum likelihood (ML) trees of ITS,** ***trnL-F*** **and** ***matK*** **, computed by RAxML with 100 bootstrap replicates,** ***H. subulifolium*** **re-sequences in this study were included.** The bootstrap values are given below the branches.
(TIF)

Figure S14 **Strict consensus tree of most parsimonious trees based on the combined datasets of ITS,** ***trnL-F*** **and** ***matK*** **,** ***H. subulifolium*** **re-sequences in this study was included.** Tree length = 1471 steps, CI = 0.7294, RI = 0.7244. The bootstrap values of the maximum parsimony analysis are given below the branches.
(TIF)

Figure S15 **Alignment of** ***trnL-F*** **: sites** ***286–377*** **.** The species name suffix with “DL” mean the sequences originate from Fan's paper.
(TIF)

Figure S16 **Alignment of** ***trnL-F*** **: sites** ***423–539*** **.** The species name suffix with “DL” mean the sequences originate from Fan's paper.
(TIF)

Figure S17 **Alignment of** ***trnL-F*** **: sites** ***611–779*** **.** The species name suffix with “DL” mean the sequences originate from Fan's paper.
(TIF)

Figure S18 **Alignment of** ***trnL-F*** **: sites** ***856–930*** **.** The species name suffix with “DL” mean the sequences originate from Fan's paper.
(TIF)

Figure S19 ***Holcoglossum*** **.** **a–c.** *H. quasipinifolium* (genus and *H*. section *Holcoglossum* type): **a.** Inflorescence; **b.** Flower, side view; **c.** Pollinarium. **d–e.** *H. nujiangense* (*H*. section *Nujiangensia* type): **d.** Inflorescence; **e.** Flower, side view. **f–g.** *H. sinicum* (*H*. section *Sorotylos* type): **f.** Inflorescence; **g.** Flower, side view.
(TIF)

Figure S20 ***Paraholcoglossum*** **.** **a–d.** *P. amesianum* (genus type): **a.** Flowers; **b.** Lip and column, side view; **c.** Pollinarium, front view; **d.** Pollinarium, back view; **e–f.** *P. subulifolium*: **e.** Flowers, front view; **f.** Flower, side view; **g–h.** *P. auriculatum*: **g.** Flower, longitudinal section; **h.** Flower, side view.
(TIF)

Figure S21 ***Tsiorchis.*** **a–d.** *T. kimballiana* (genus type): **a.** Flowers; **b.** Lip and column, side view; **c.** Pollinarium, front view; **d.** Pollinarium, back view. **e–h.** *T. wangii*: **e.** Inflorescence; **f.** Flower, side view; **g.** Pollinarium, front view; **h.** Pollinia, back view.
(TIF)

Figure S22 ***Holcoglossum linearifolium.*** **a.** Natural habitat in SW Yunnan; **b.** Growing on tree trunk; **c, d.** Flowers.
(TIF)

Morphological character codes S1 (DOC)

Table S1 Pair-wise genetic distance of all species based on combined datasets of ITS, *trnL-F*, and *matK*.
(DOC)

Table S2 Samples used in the gene sequencing and their information.
(DOC)

Table S3 Genetic distances between sections of the *Holcoglossum* clade.
(DOC)

Table S4 Morphological data matrix for the phylogenetic analysis.
(DOC)
