# Supplementary figures and images for: Paraholcoglossum and Tsiorchis, Two New Orchid Genera Established by Molecular and Morphological Analyses of the Holcoglossum Alliance
Source: PLoS One. 2011 Oct 10;6(10):e24864. doi: 10.1371/journal.pone.0024864 (PMC3189912; doi:10.1371/journal.pone.0024864)

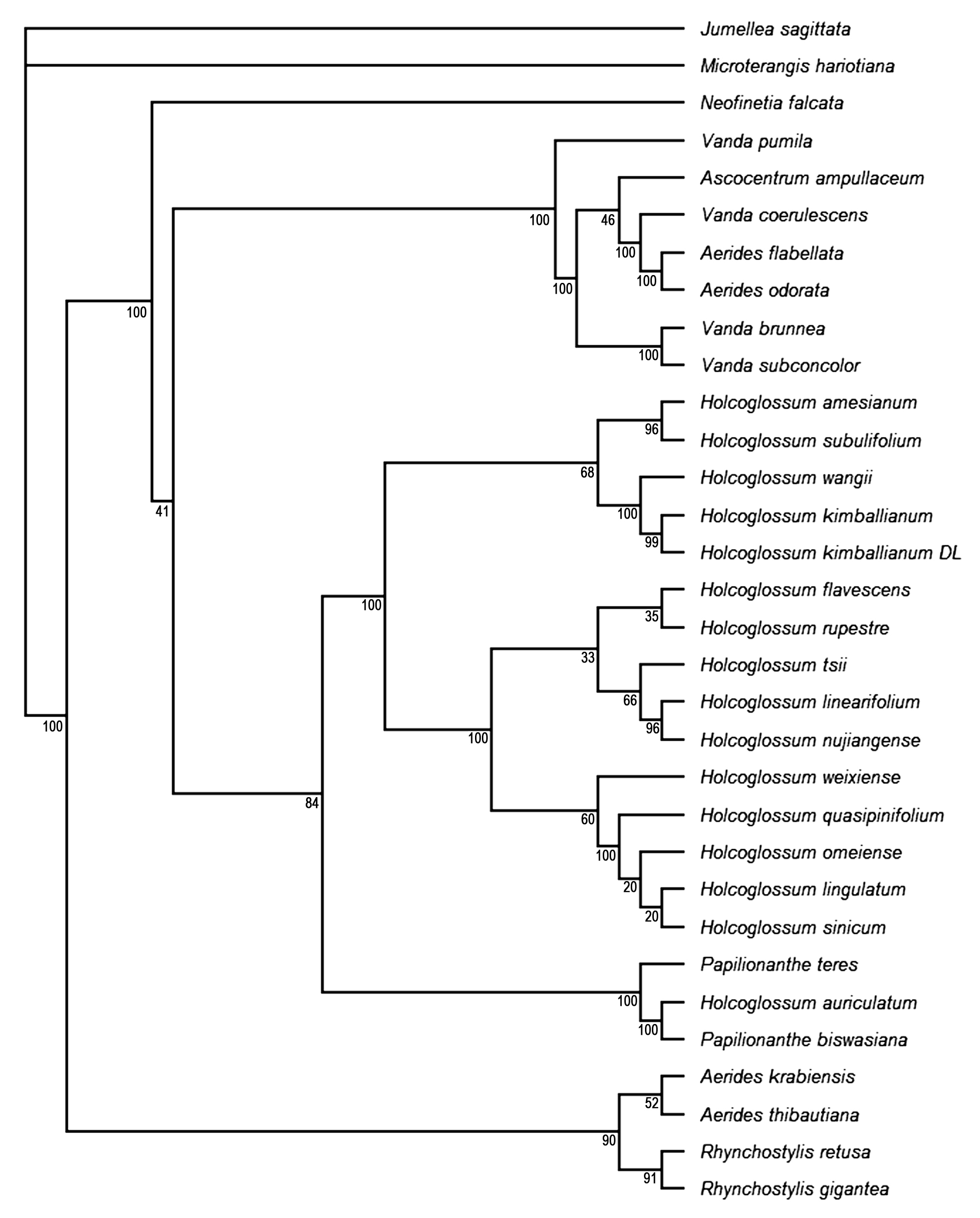

Supplement: Figure S1 — Bayesian consensus trees based on the last 30,001 maximum likelihood trees for ITS. The Bayesian posterior probability (×100) is given below the branches. (TIF) [file pone.0024864.s001.tif]

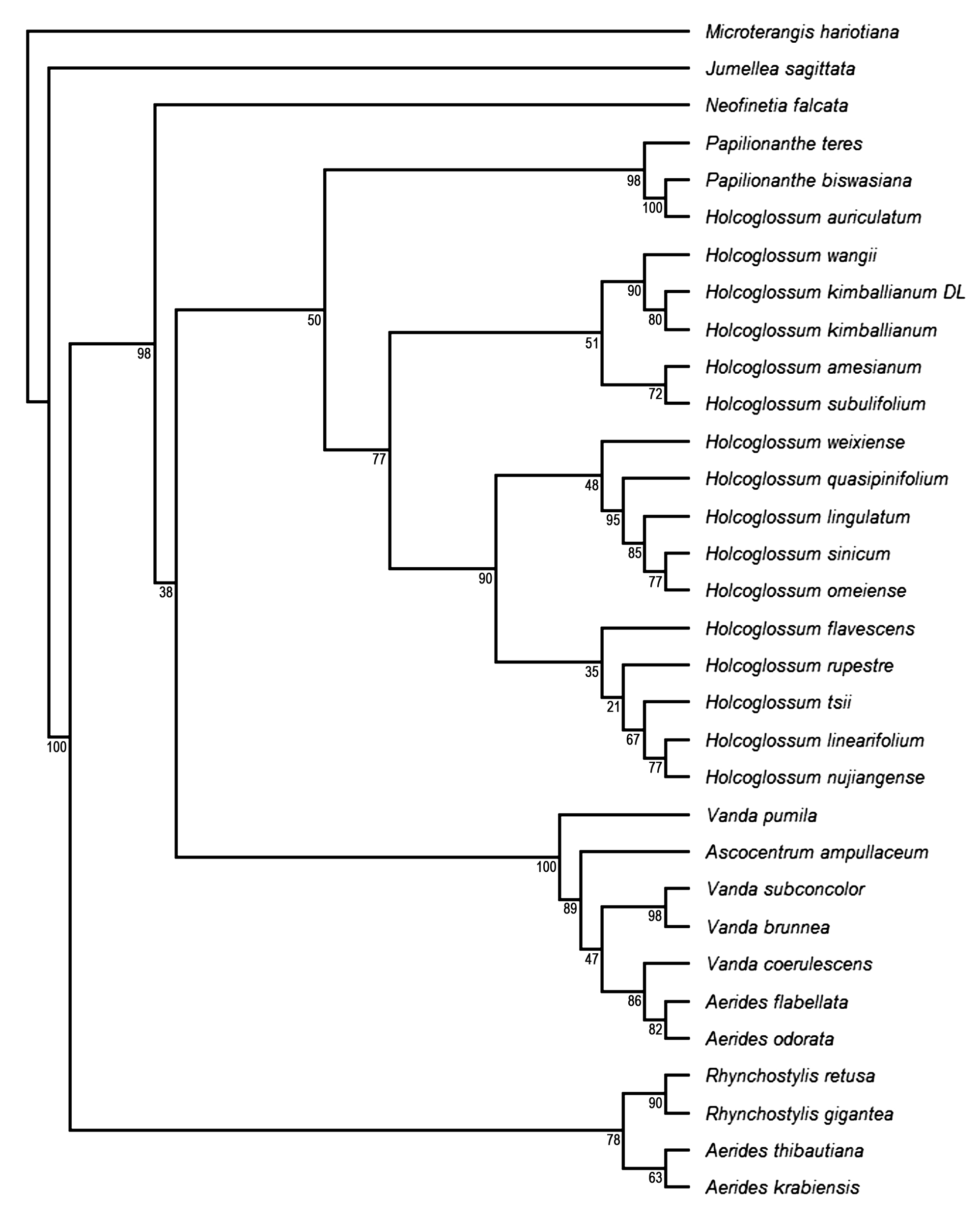

Supplement: Figure S2 — The maximum likelihood (ML) trees of ITS computed by RAxML with 100 bootstrap replicates. The bootstrap values are given below the branches. (TIF) [file pone.0024864.s002.tif]

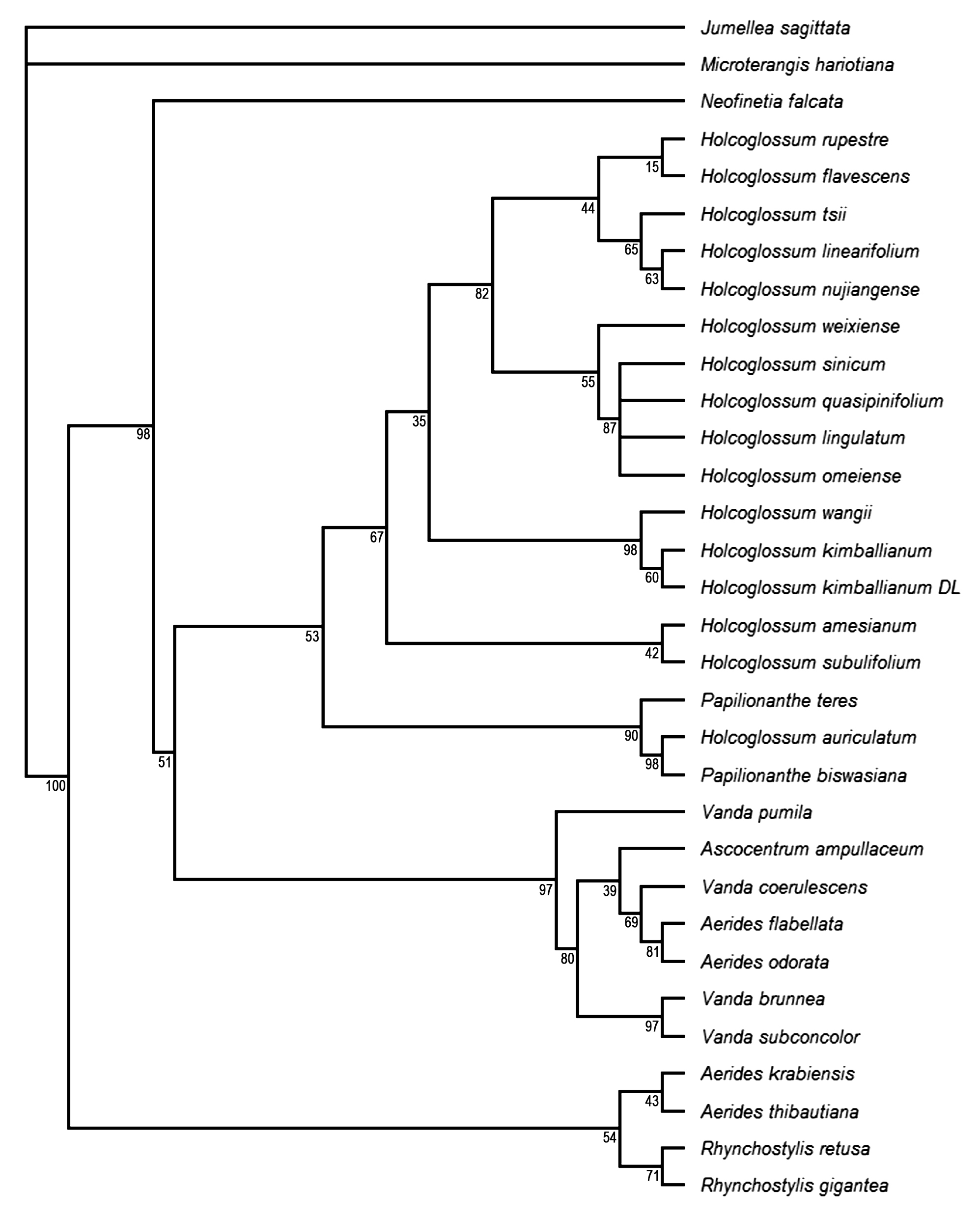

Supplement: Figure S3 — Strict consensus tree of most parsimonious trees based on of ITS sequence data. Tree length = 347 steps, CI = 0.6801, RI = 0.7511. The bootstrap values of the maximum parsimony analysis are given below the branches. (TIF) [file pone.0024864.s003.tif]

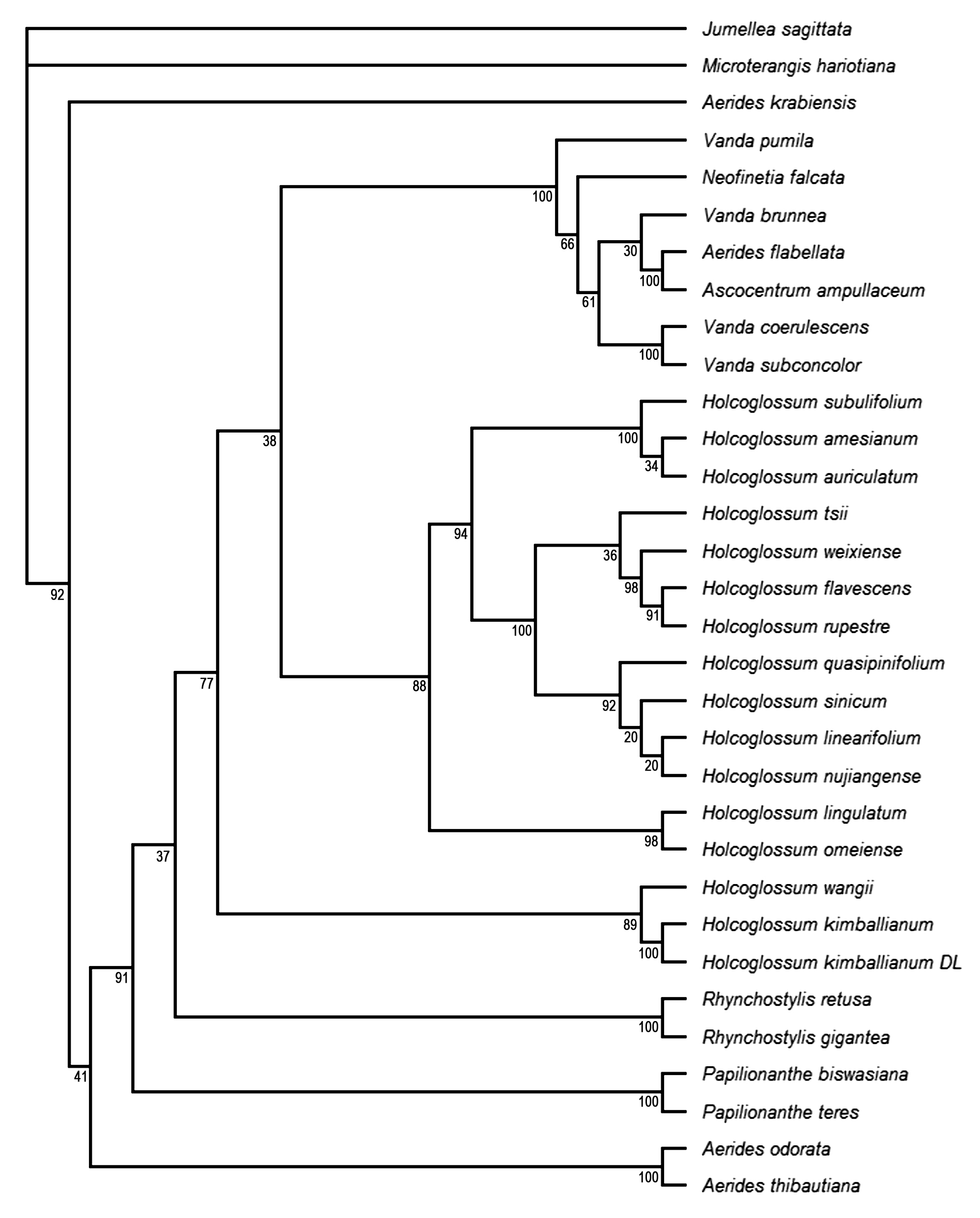

Supplement: Figure S4 — Bayesian consensus trees based on the last 30,001 maximum likelihood trees for trnL-F . The Bayesian posterior probability (×100) is given below the branches. (TIF) [file pone.0024864.s004.tif]

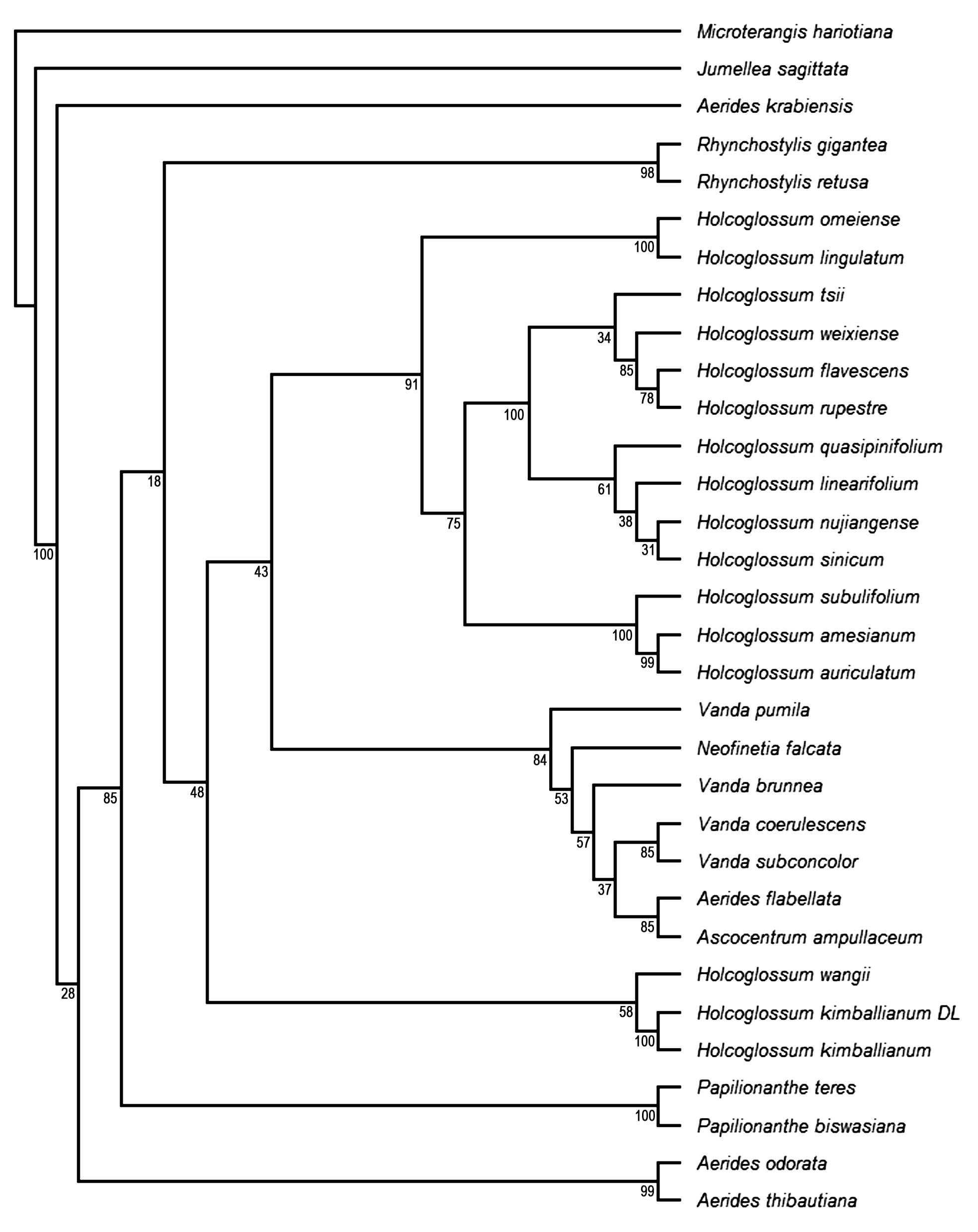

Supplement: Figure S5 — The maximum likelihood (ML) trees of trnL-F , computed by RAxML with 100 bootstrap replicates. The bootstrap values are given below the branches. (TIF) [file pone.0024864.s005.tif]

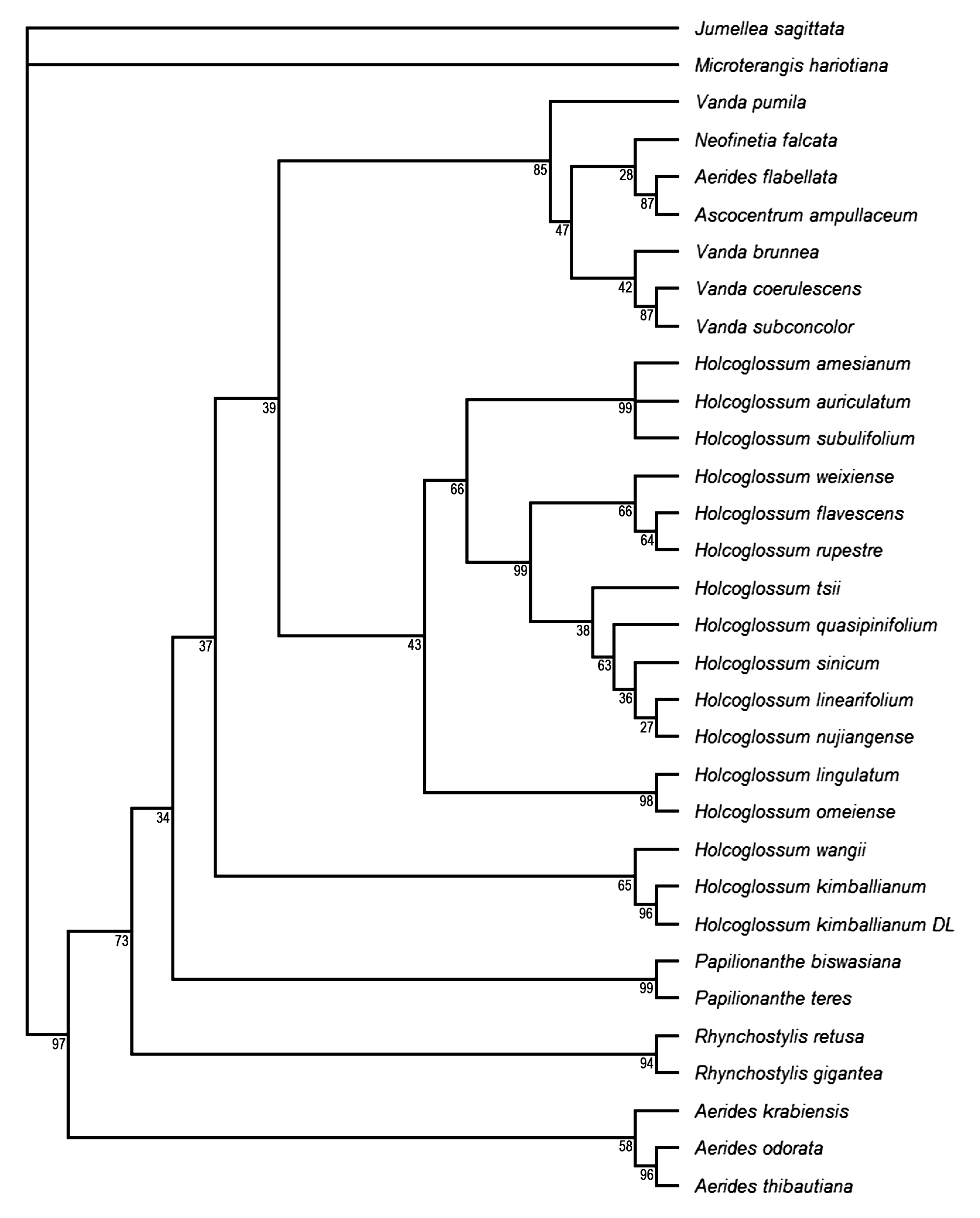

Supplement: Figure S6 — Strict consensus tree of most parsimonious trees based on of trnL-F sequence data. Tree length = 442 steps, CI = 0.8348, RI = 0.8685. The bootstrap values of the maximum parsimony analysis are given below the branches. (TIF) [file pone.0024864.s006.tif]

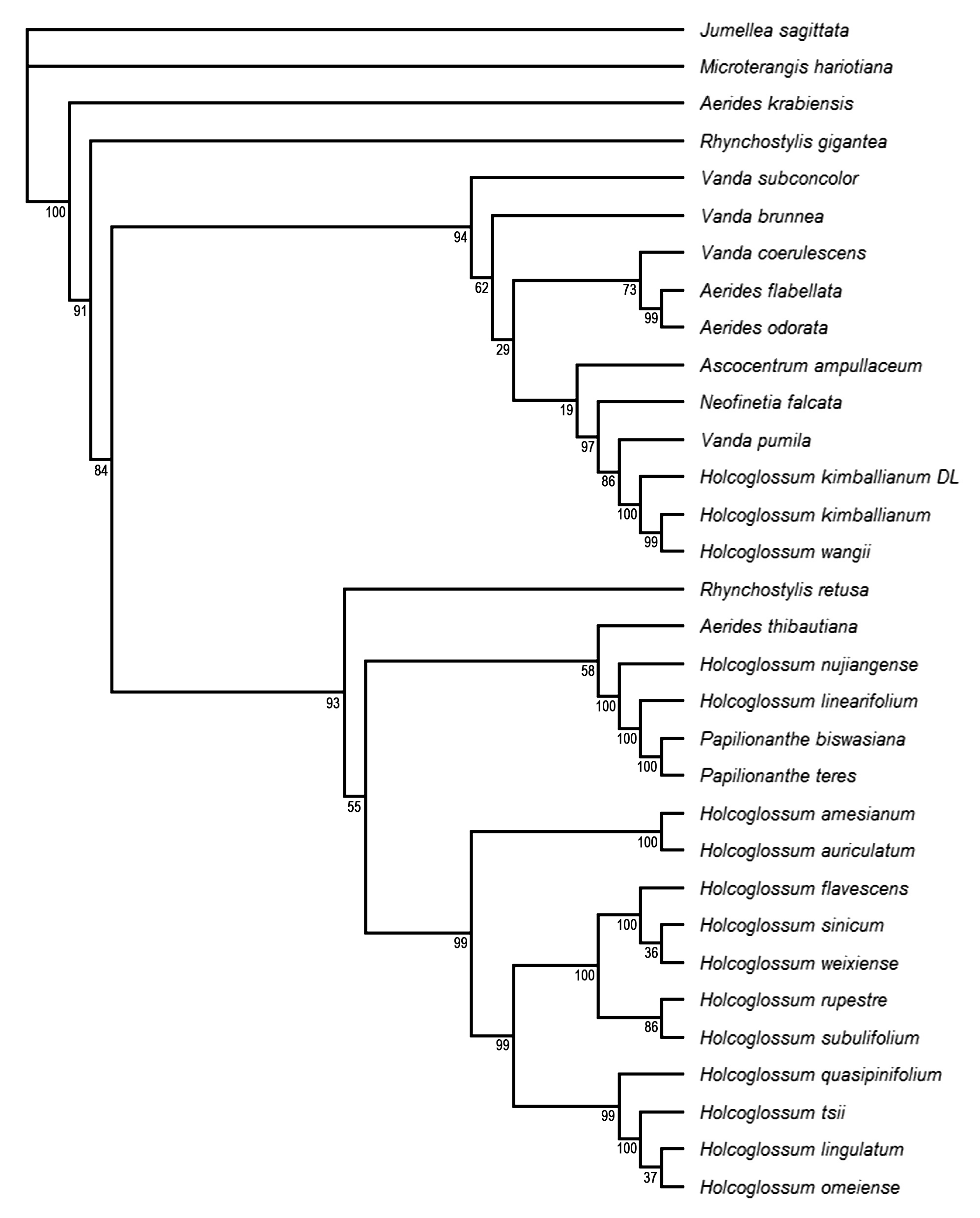

Supplement: Figure S7 — Bayesian consensus trees based on the last 30,001 maximum likelihood trees for matK . The Bayesian posterior probability (×100) is given below the branches. (TIF) [file pone.0024864.s007.tif]

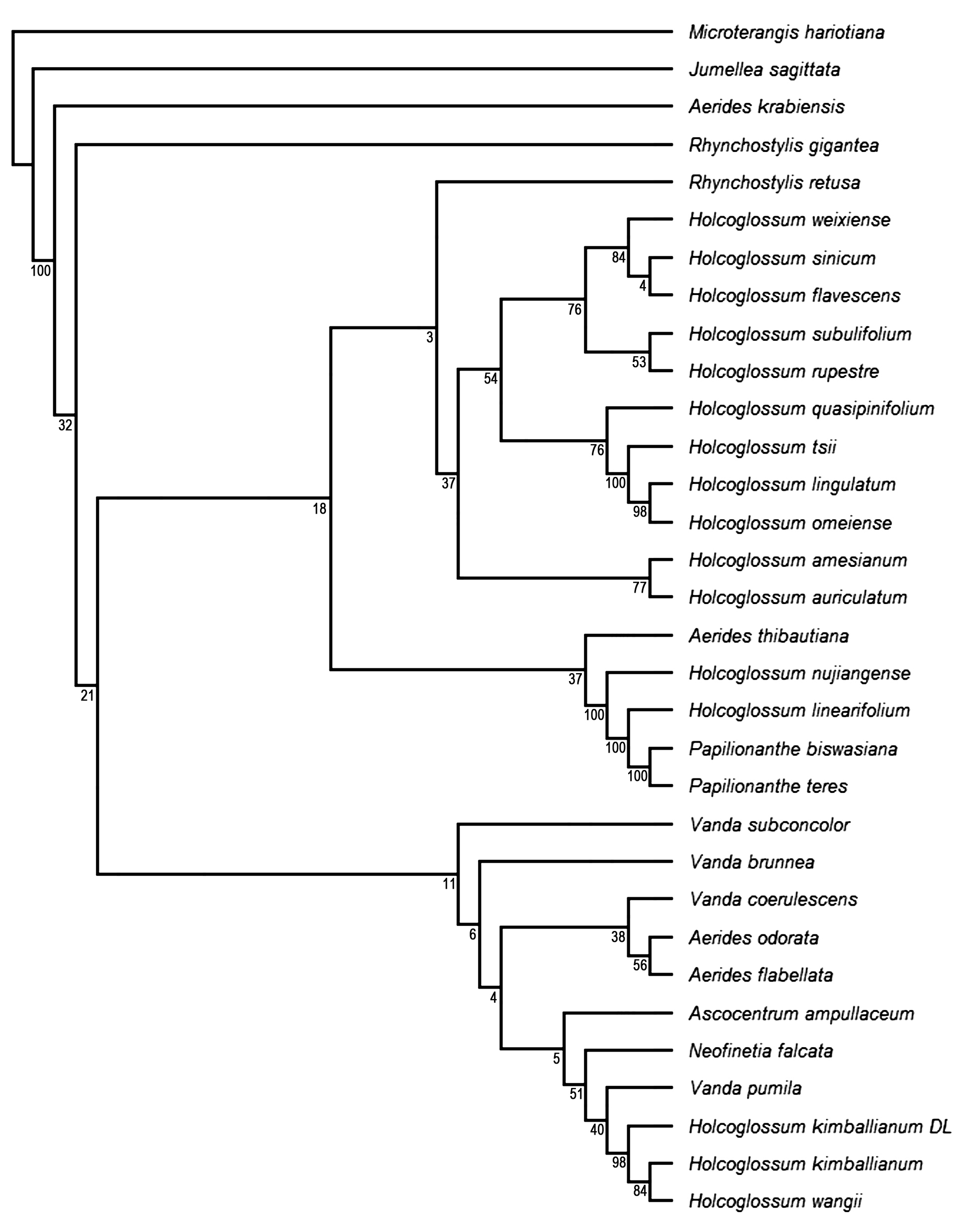

Supplement: Figure S8 — The maximum likelihood (ML) trees of matK , computed by RAxML with 100 bootstrap replicates. The bootstrap values are given below the branches. (TIF) [file pone.0024864.s008.tif]

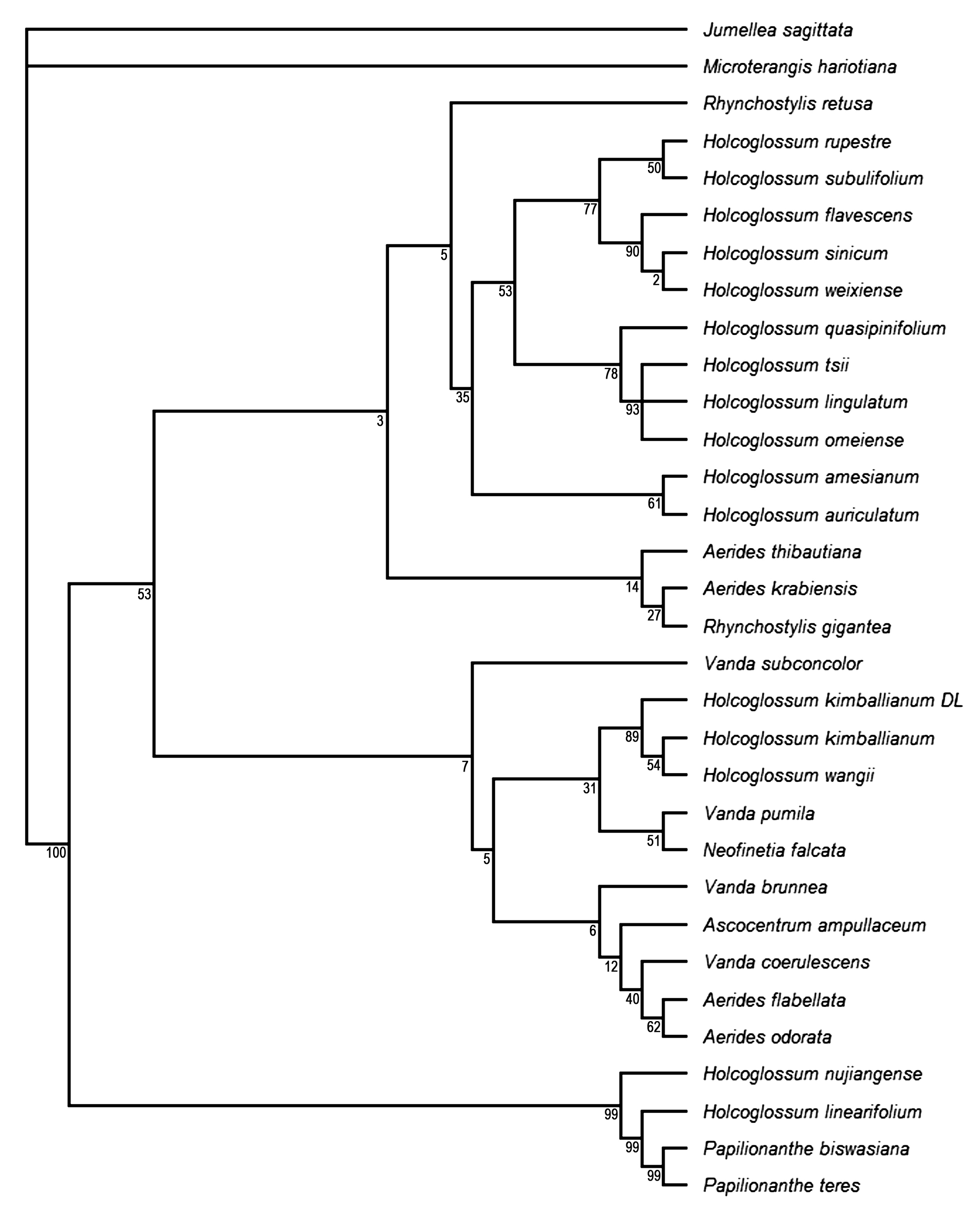

Supplement: Figure S9 — Strict consensus tree of most parsimonious trees based on matK sequence data. Tree length = 552 steps, CI = 0.8333, RI = 0.7870. The bootstrap values of the maximum parsimony analysis are given below the branches. (TIF) [file pone.0024864.s009.tif]

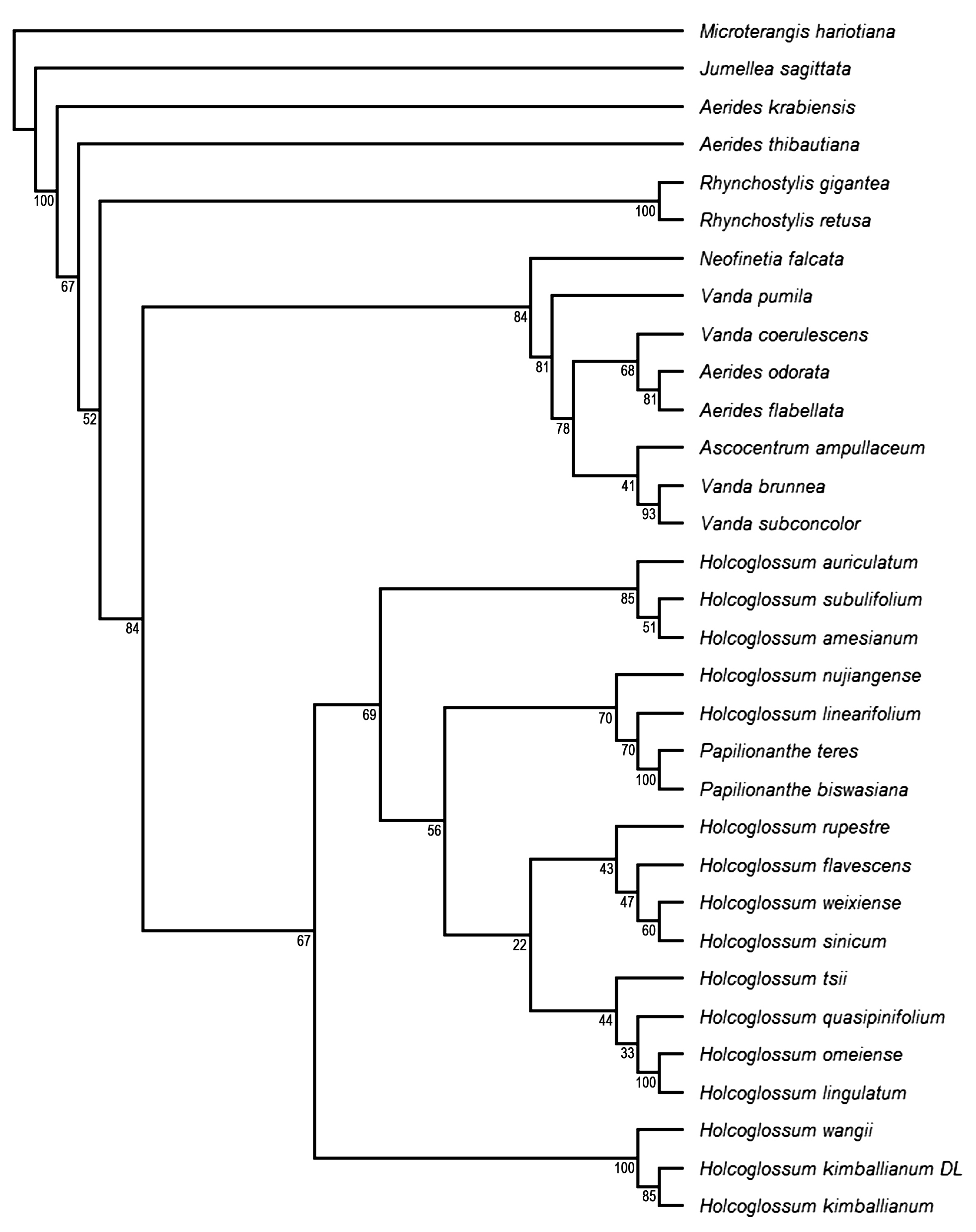

Supplement: Figure S10 — The maximum likelihood (ML) trees of ITS, trnL-F and matK , computed by RAxML with 100 bootstrap replicates. The bootstrap values of are given below the branches. (TIF) [file pone.0024864.s010.tif]

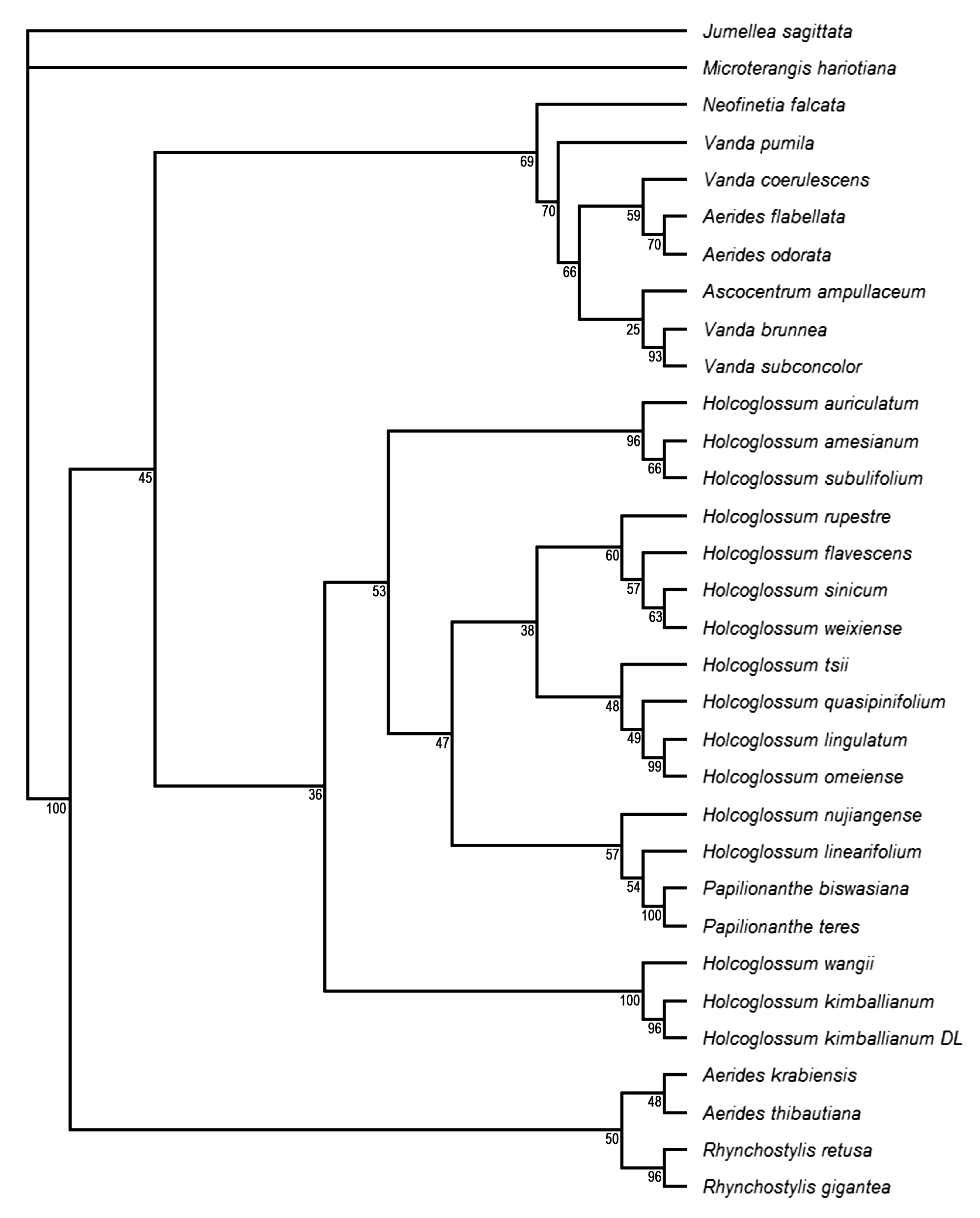

Supplement: Figure S11 — Strict consensus tree of most parsimonious trees based on the combined datasets of ITS, trnL-F and matK . Tree length = 1455 steps, CI = 0.7331, RI = 0.7216. The bootstrap values of the maximum parsimony analysis are given below the branches. (TIF) [file pone.0024864.s011.tif]

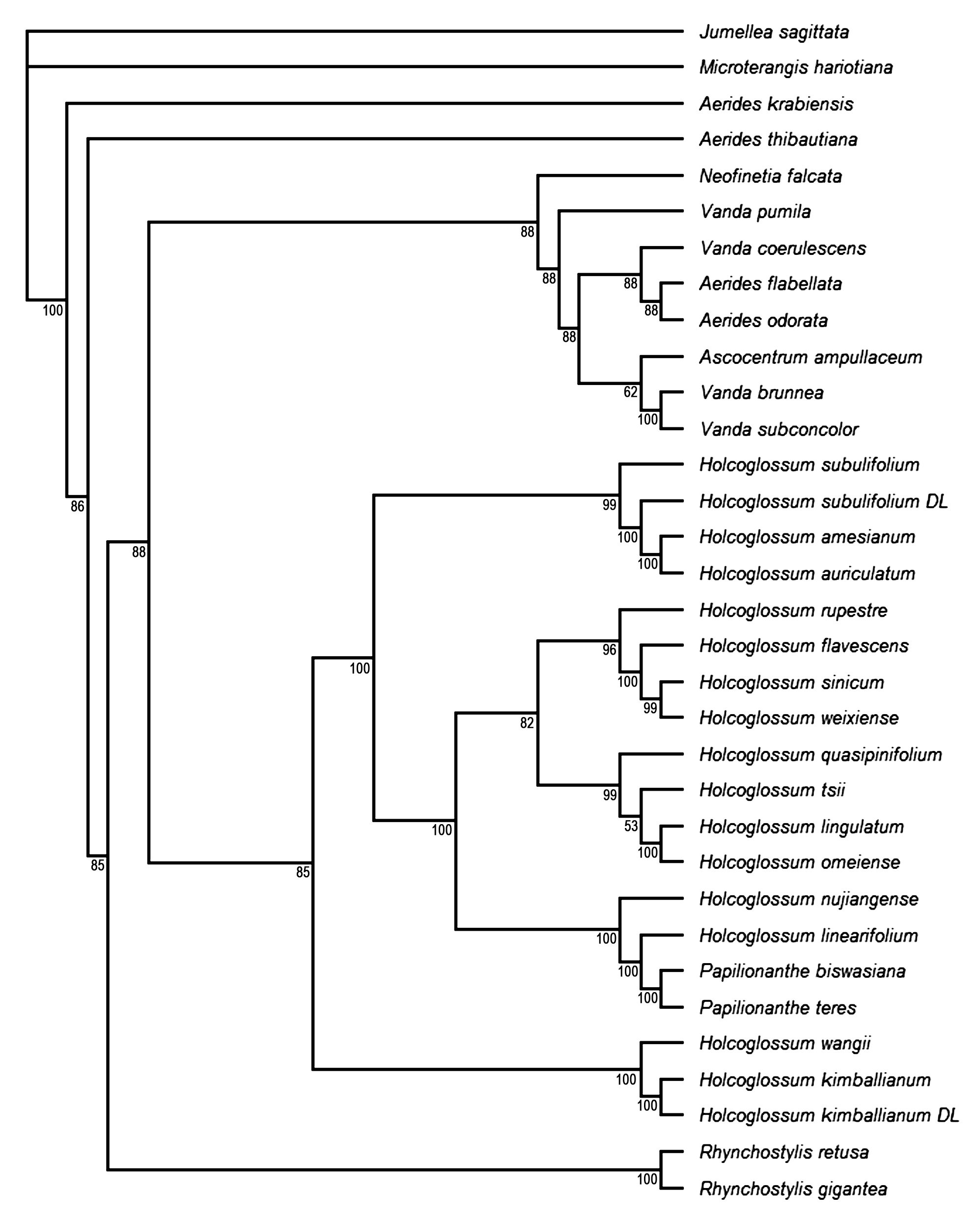

Supplement: Figure S12 — Bayesian consensus trees based on the last 30,001 maximum likelihood trees for ITS, trnL-F and matK , H. subulifolium re-sequences in this study was included. The Bayesian posterior probability (×100) is given below the branches. (TIF) [file pone.0024864.s012.tif]

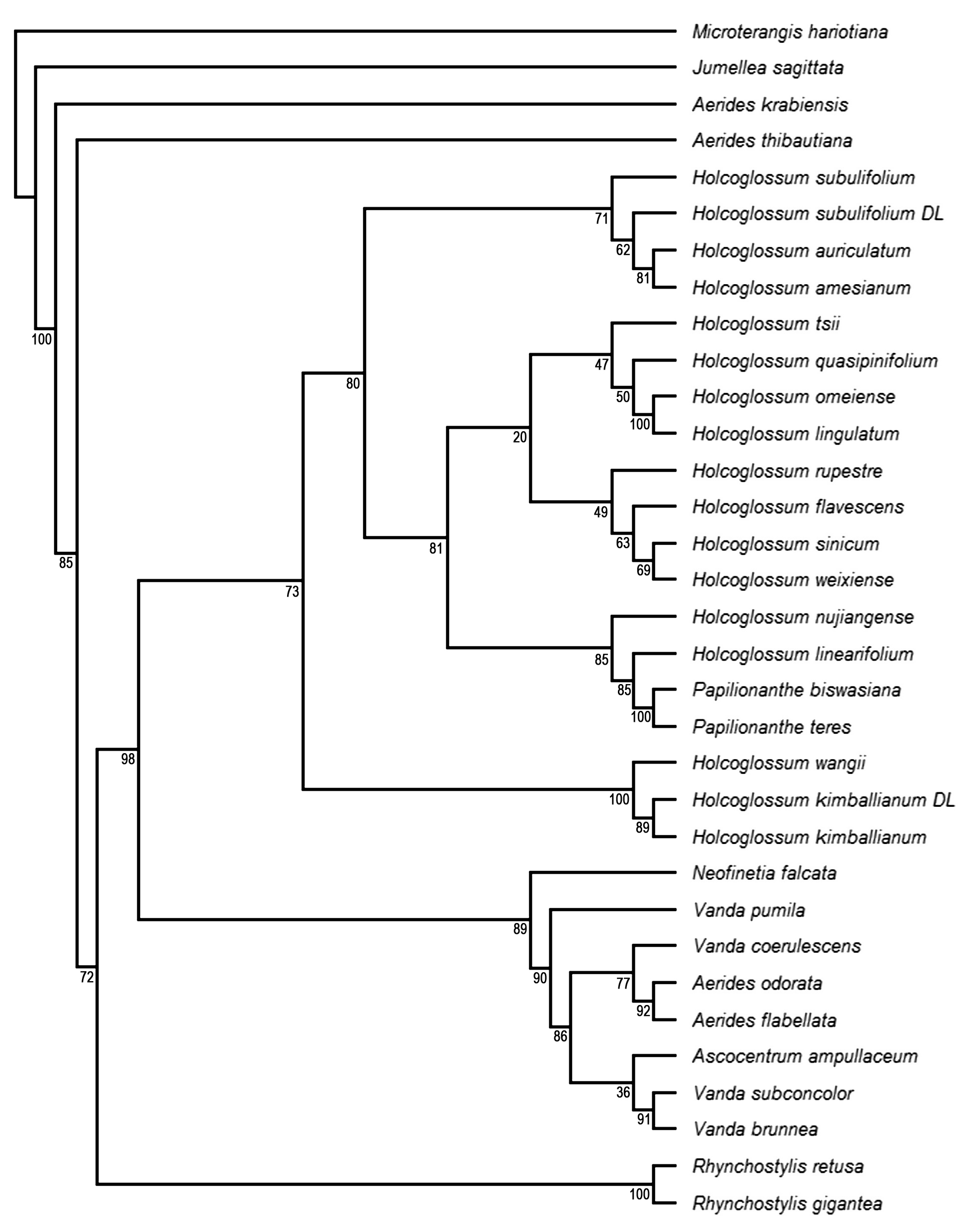

Supplement: Figure S13 — The maximum likelihood (ML) trees of ITS, trnL-F and matK , computed by RAxML with 100 bootstrap replicates, H. subulifolium re-sequences in this study were included. The bootstrap values are given below the branches. (TIF) [file pone.0024864.s013.tif]

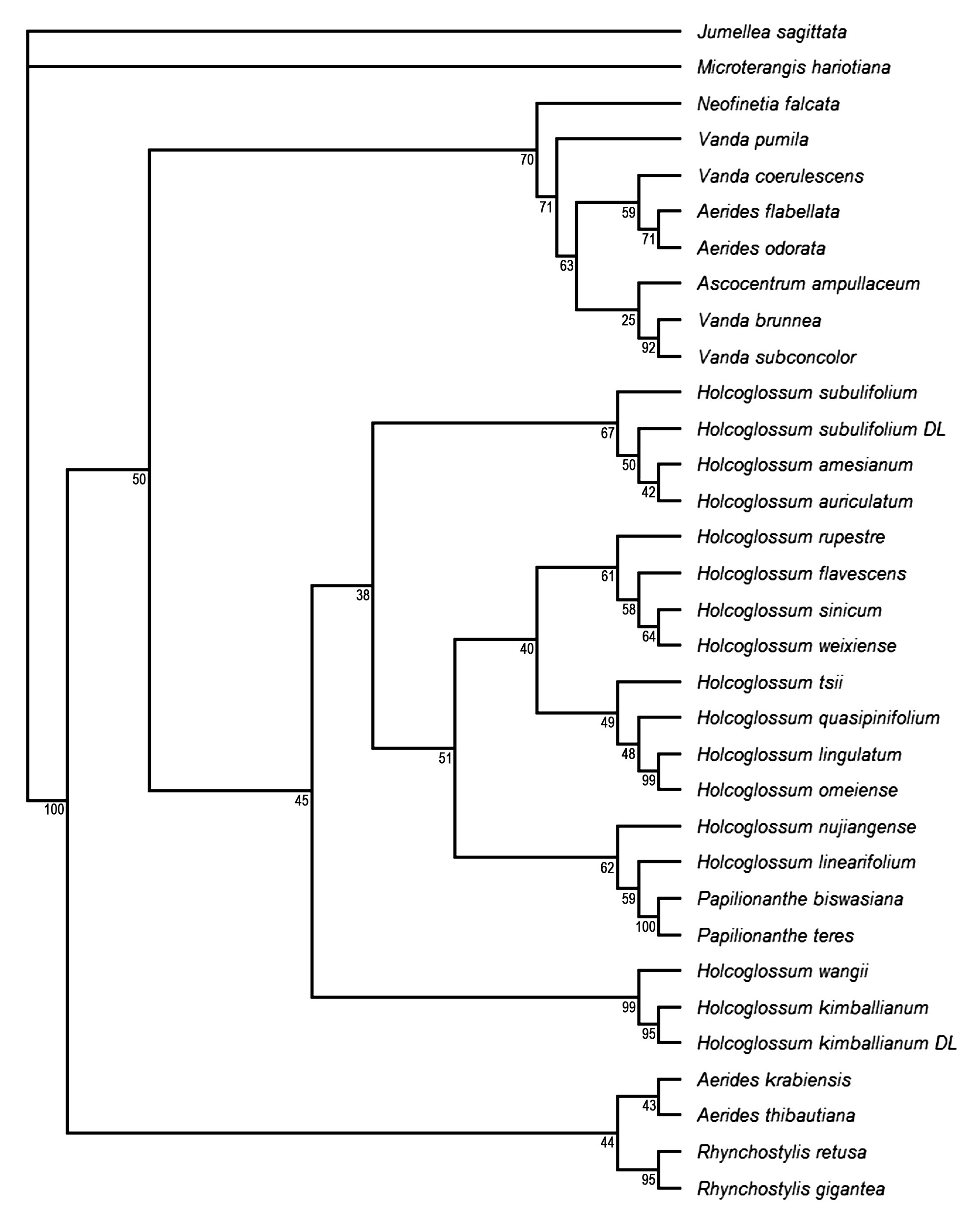

Supplement: Figure S14 — Strict consensus tree of most parsimonious trees based on the combined datasets of ITS, trnL-F and matK , H. subulifolium re-sequences in this study was included. Tree length = 1471 steps, CI = 0.7294, RI = 0.7244. The bootstrap values of the maximum parsimony analysis are given below the branches. (TIF) [file pone.0024864.s014.tif]

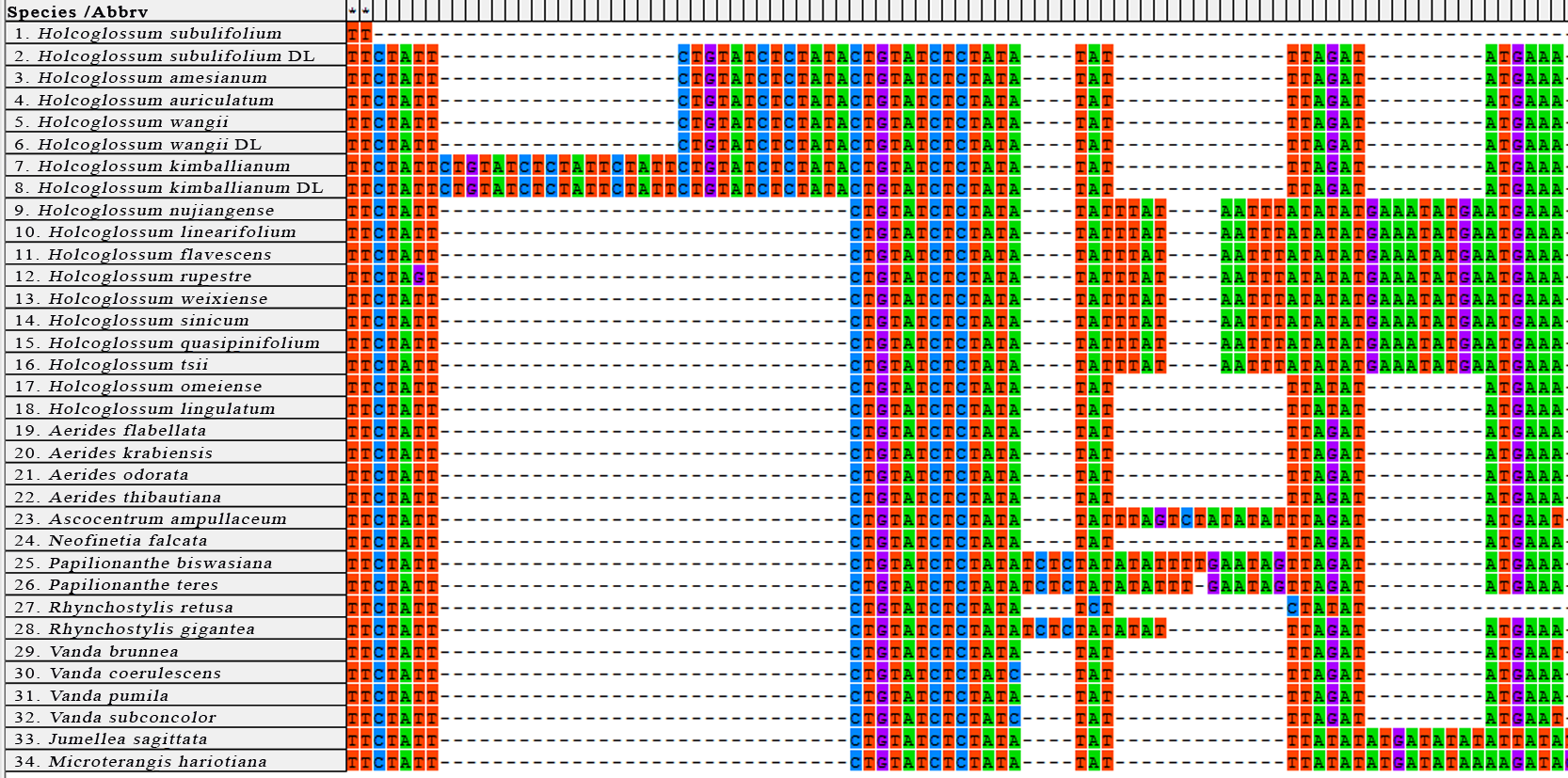

Supplement: Figure S15 — Alignment of trnL-F : sites 286–377 . The species name suffix with “DL” mean the sequences originate from Fan's paper. (TIF) [file pone.0024864.s015.tif]

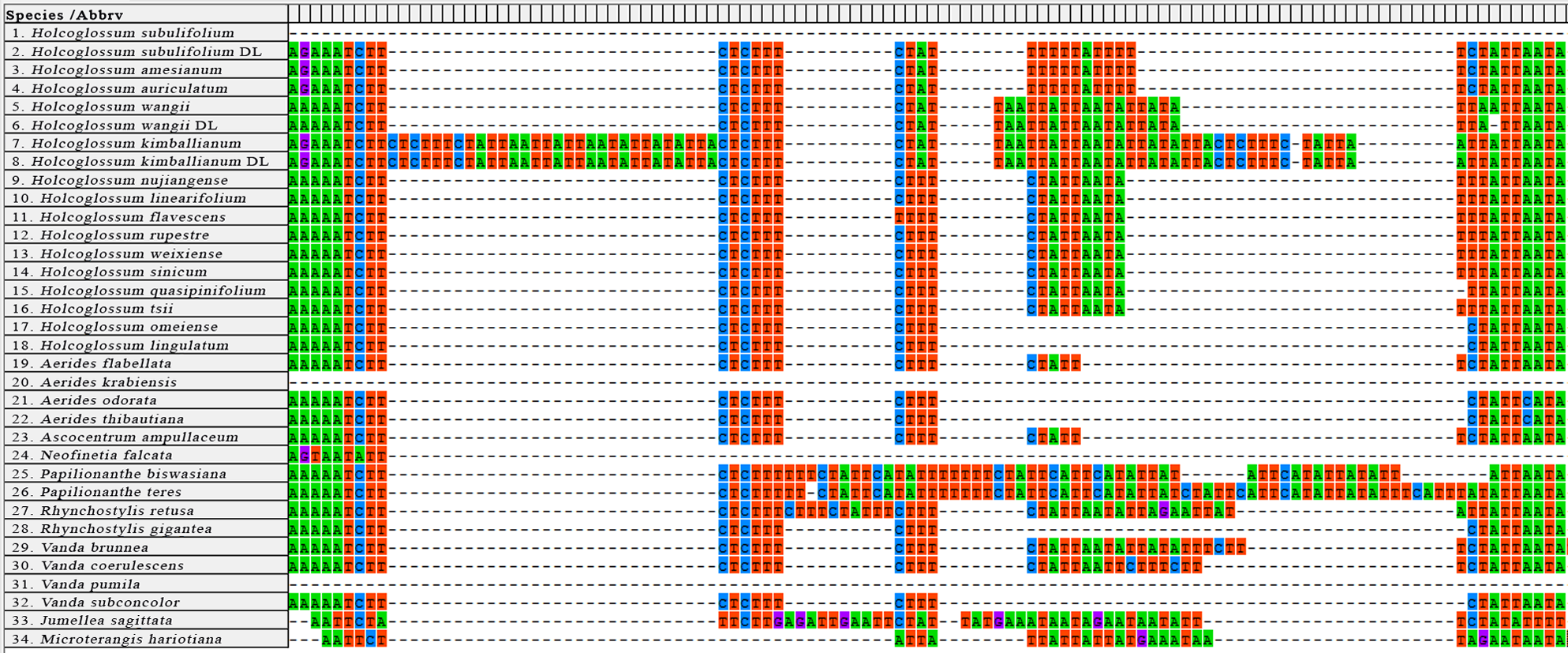

Supplement: Figure S16 — Alignment of trnL-F : sites 423–539 . The species name suffix with “DL” mean the sequences originate from Fan's paper. (TIF) [file pone.0024864.s016.tif]

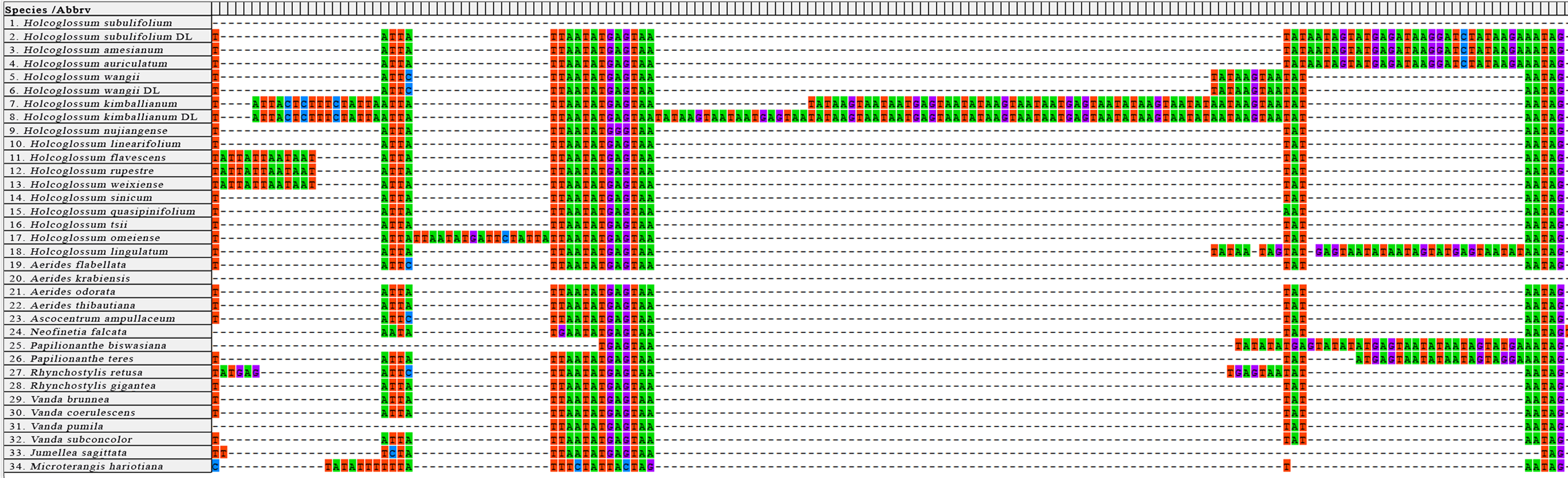

Supplement: Figure S17 — Alignment of trnL-F : sites 611–779 . The species name suffix with “DL” mean the sequences originate from Fan's paper. (TIF) [file pone.0024864.s017.tif]

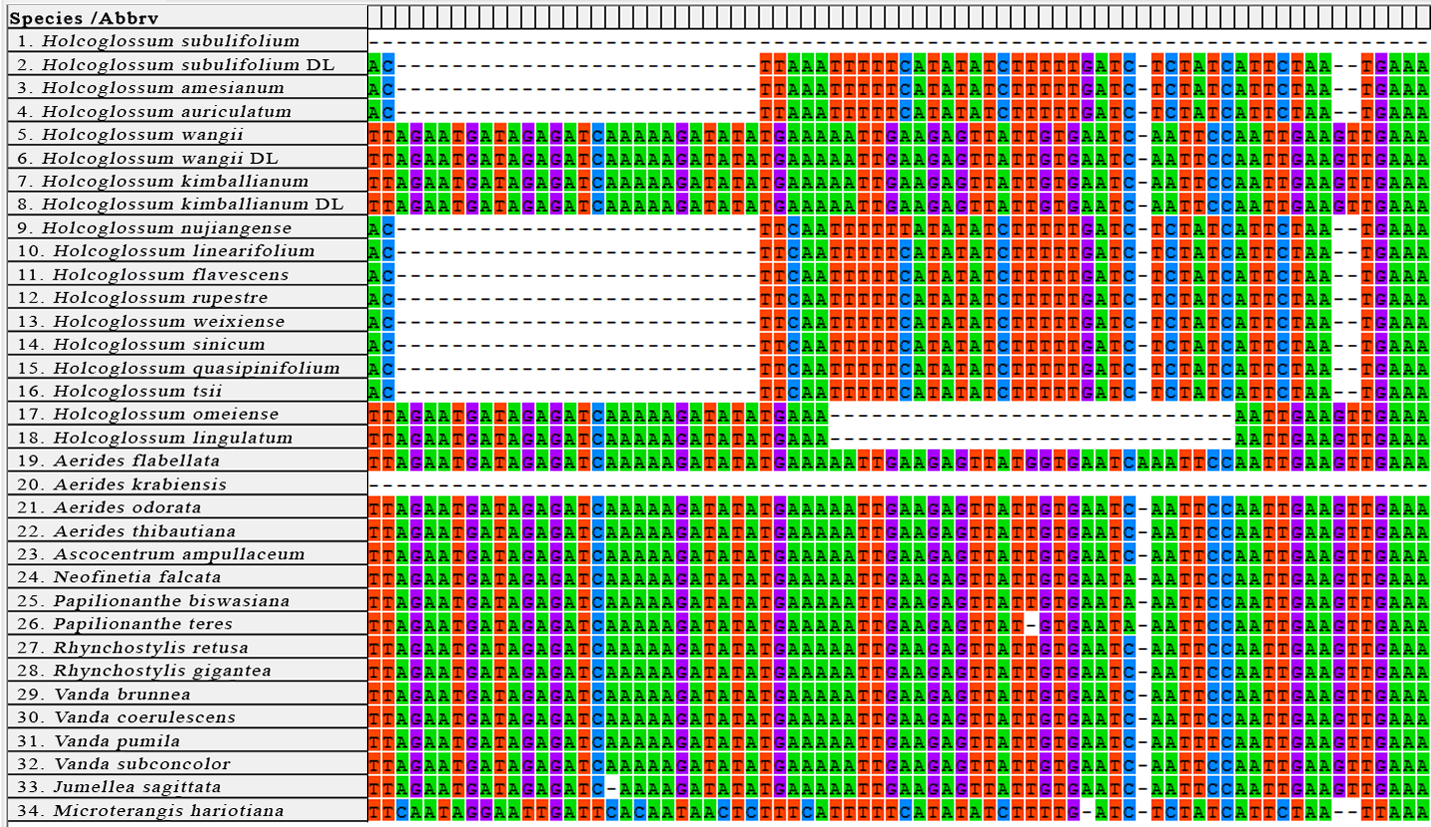

Supplement: Figure S18 — Alignment of trnL-F : sites 856–930 . The species name suffix with “DL” mean the sequences originate from Fan's paper. (TIF) [file pone.0024864.s018.tif]

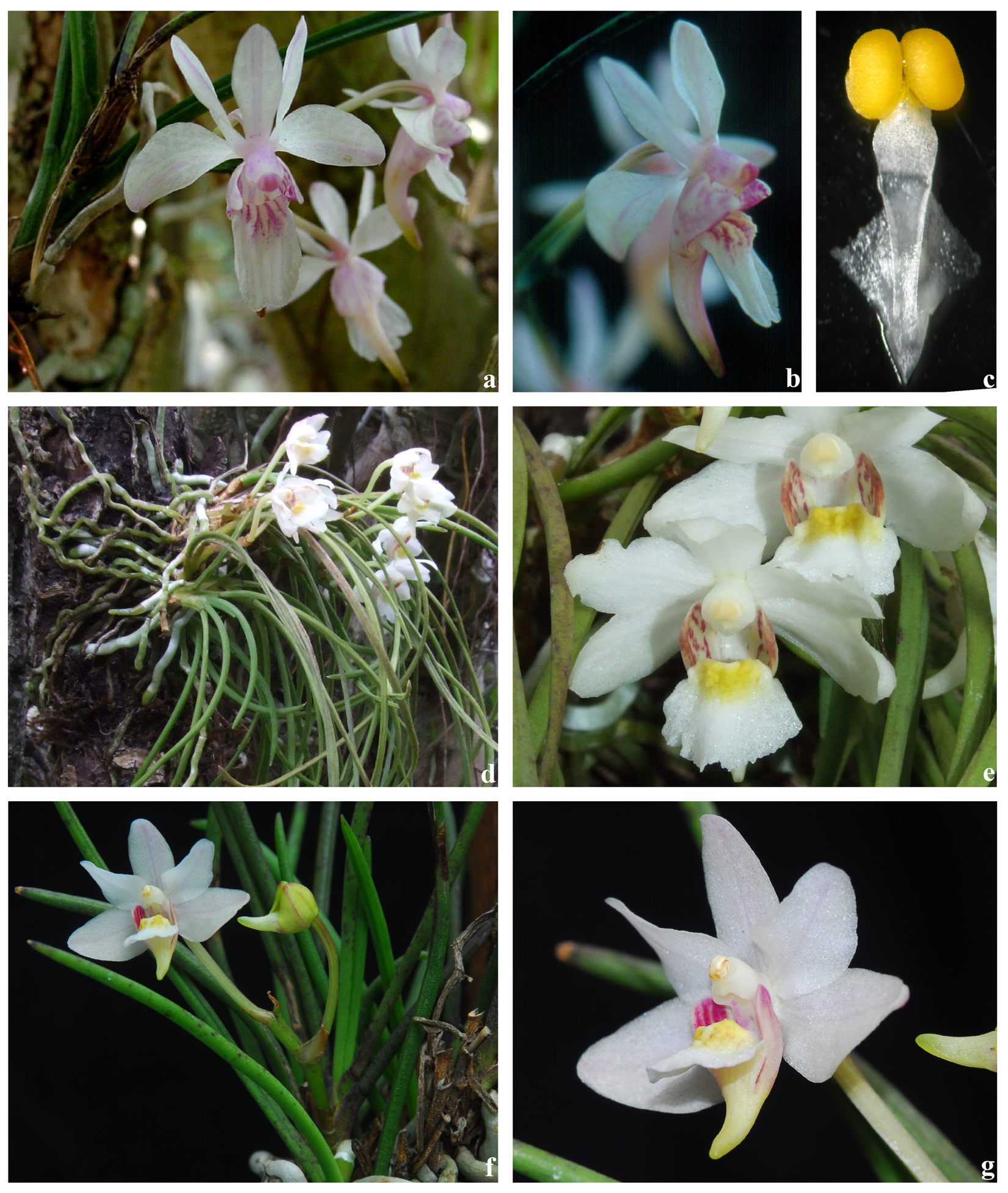

Supplement: Figure S19 — Holcoglossum . a–c. H. quasipinifolium (genus and H. section Holcoglossum type): a. Inflorescence; b. Flower, side view; c. Pollinarium. d–e. H. nujiangense (H. section Nujiangensia type): d. Inflorescence; e. Flower, side view. f–g. H. sinicum (H. section Sorotylos type): f. Inflorescence; g. Flower, side view. (TIF) [file pone.0024864.s019.tif]

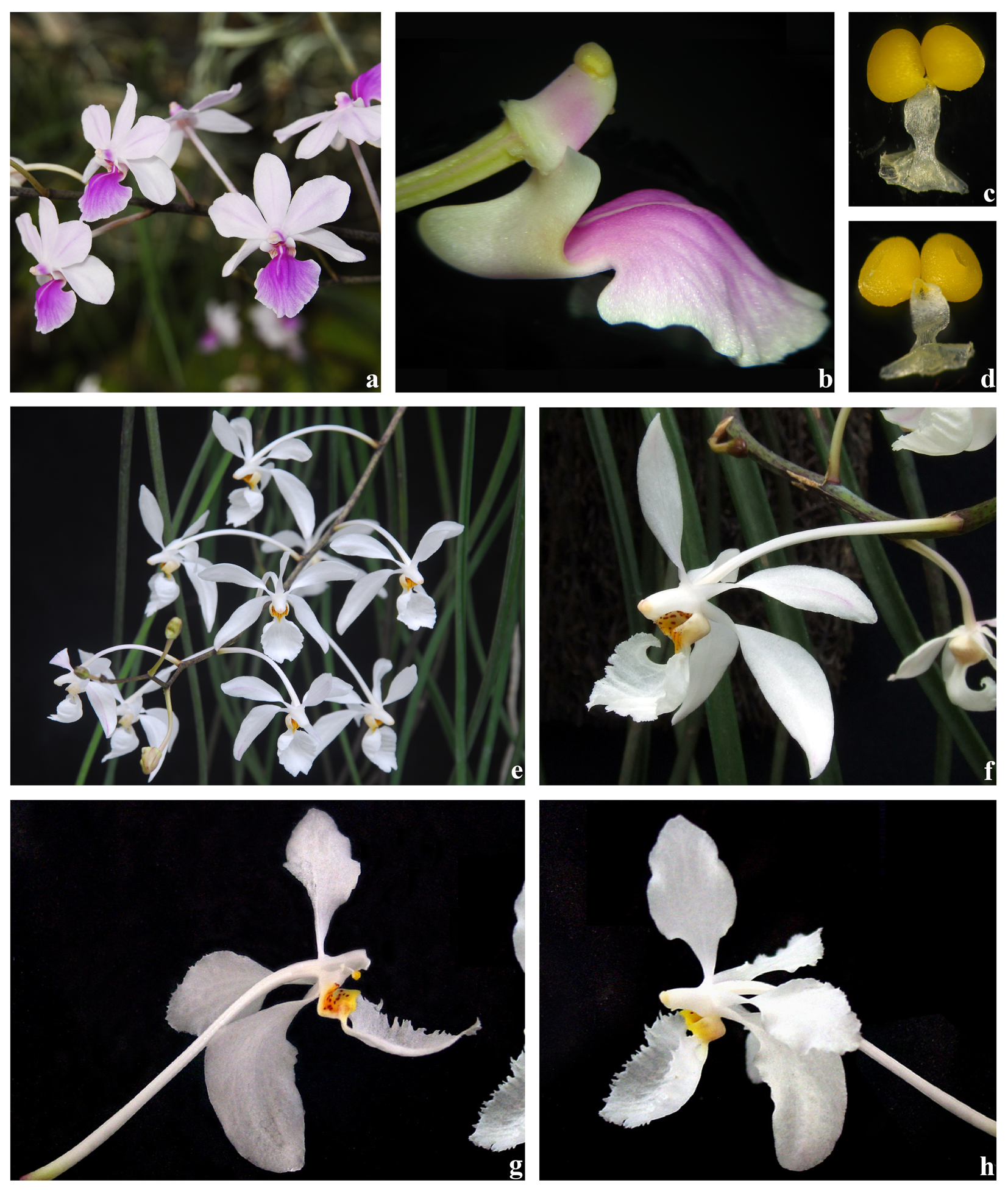

Supplement: Figure S20 — Paraholcoglossum . a–d. P. amesianum (genus type): a. Flowers; b. Lip and column, side view; c. Pollinarium, front view; d. Pollinarium, back view; e–f. P. subulifolium: e. Flowers, front view; f. Flower, side view; g–h. P. auriculatum: g. Flower, longitudinal section; h. Flower, side view. (TIF) [file pone.0024864.s020.tif]

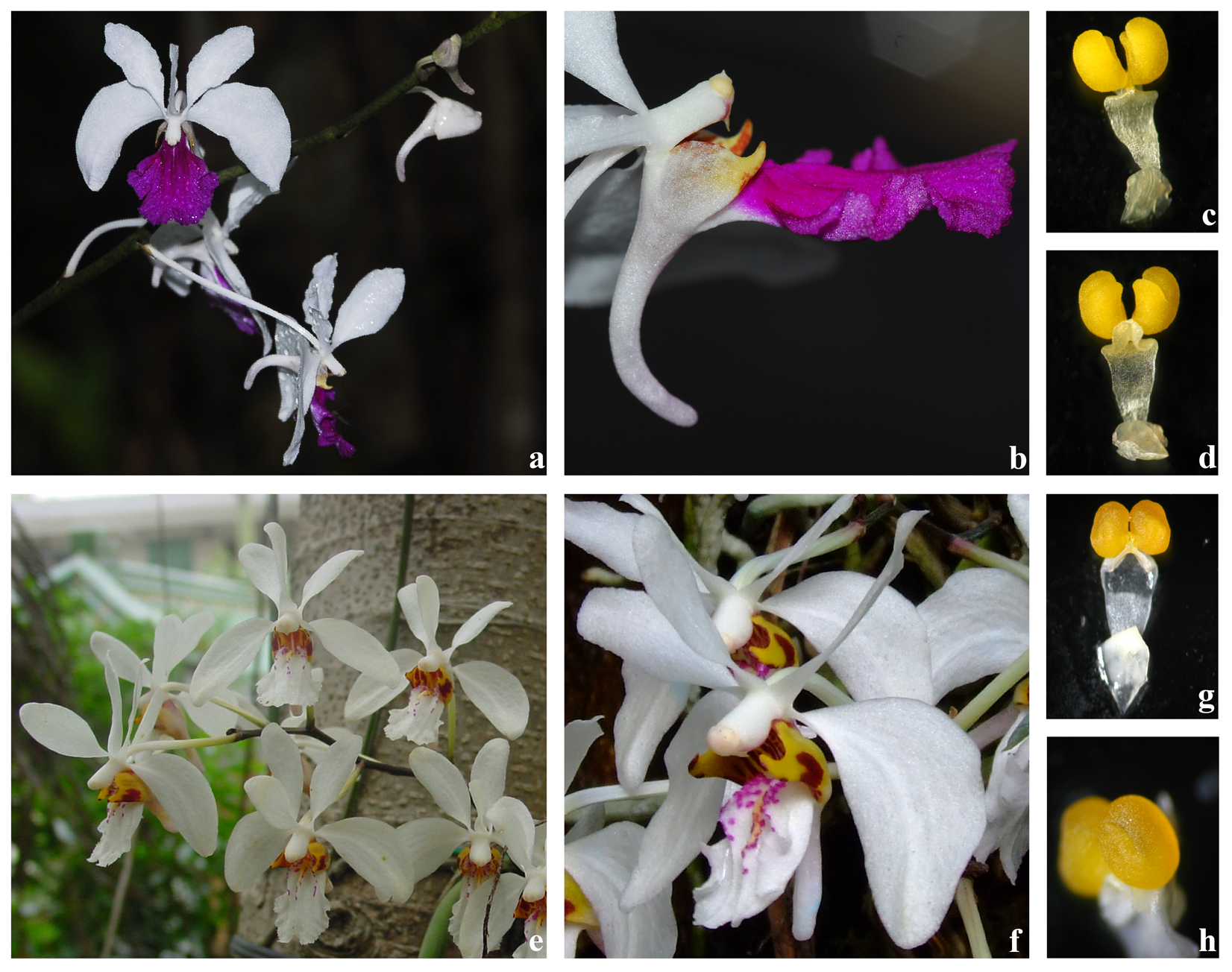

Supplement: Figure S21 — Tsiorchis. a–d. T. kimballiana (genus type): a. Flowers; b. Lip and column, side view; c. Pollinarium, front view; d. Pollinarium, back view. e–h. T. wangii: e. Inflorescence; f. Flower, side view; g. Pollinarium, front view; h. Pollinia, back view. (TIF) [file pone.0024864.s021.tif]

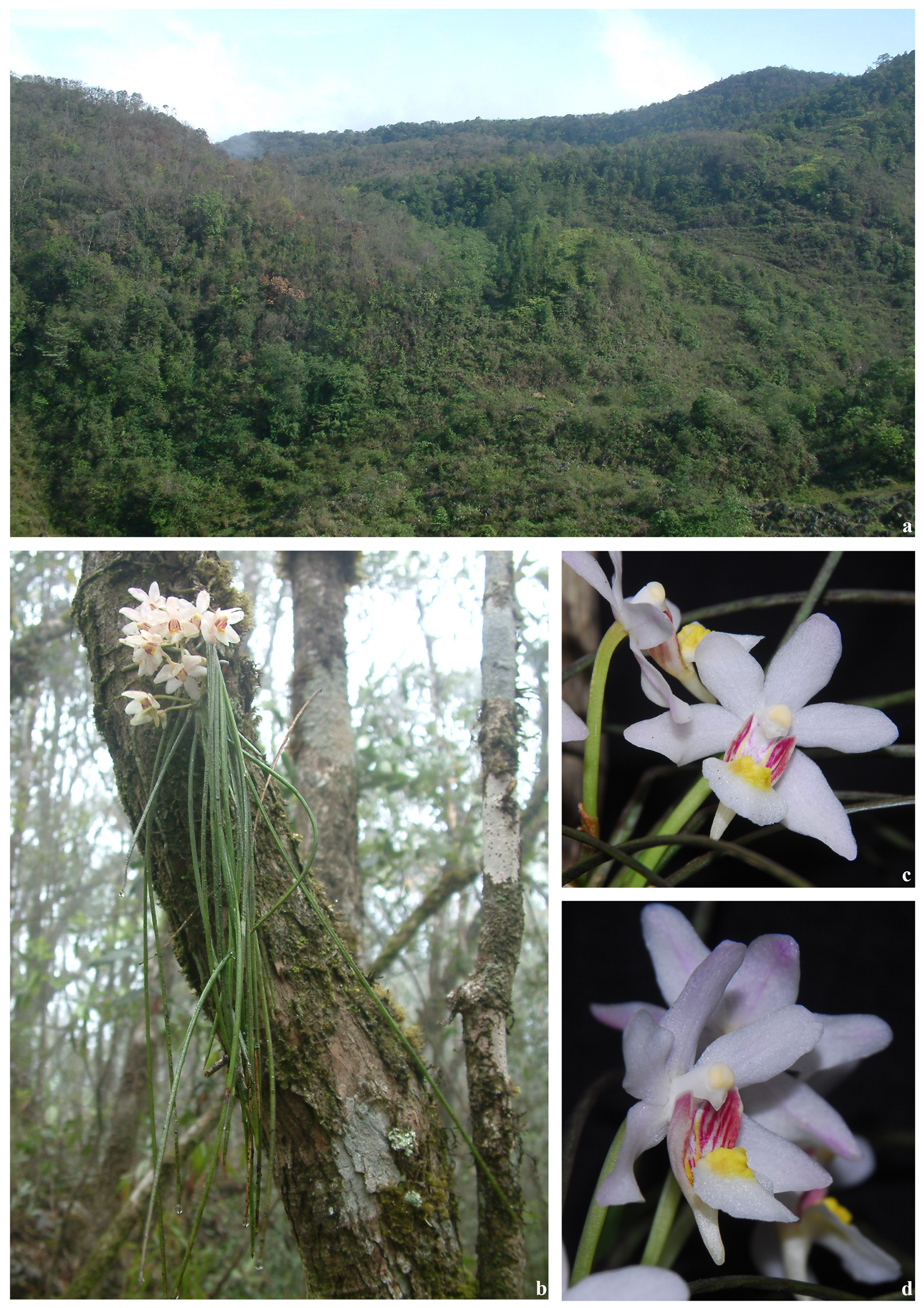

Supplement: Figure S22 — Holcoglossum linearifolium. a. Natural habitat in SW Yunnan; b. Growing on tree trunk; c, d. Flowers. (TIF) [file pone.0024864.s022.tif]
