# Supplementary material for: Paraholcoglossum and Tsiorchis, Two New Orchid Genera Established by Molecular and Morphological Analyses of the Holcoglossum Alliance
Source: PLoS One. 2011 Oct 10;6(10):e24864. doi: 10.1371/journal.pone.0024864 (PMC3189912; doi:10.1371/journal.pone.0024864)
Supplement: Morphological character codes S1 — (DOC) [file pone.0024864.s023.doc]

**Supplementary Information for**

***Paraholcoglossum* and *Tsiorchis,* Two New Orchid Genera Established By Molecular and Morphological Analyses of the *Holcoglossum* Alliance**

Zhong-Jian Liu, Li-Jun Chen, Sing-Chi Chen, Jing Cai, Wen-Chieh Tsai,

Yu-Yun Hsiao, Wen-Hui Rao, Xue-Yong Ma, Guo-Qiang Zhang

The PDF file including

Morphological character codes S1

Tables S1 to S4

Figures S1 to S22

**Morphological character codes S1**

1. root epidermis 0=rhizodermis, 1=velamen

2. growth pattern 0=sympodial, 1=monopodial

3. phyllotaxy 0=spiral, 1=distichous

4. leaf morphology 0=flat, 1=terete, as wide as its own thick, 2=broadly subterete, 2-3times wider than thick

5. winter leaf 0=absent, 1=present

6. leaf articulation 0=absent, 1=present

7. inflorescence position 0=terminal, 1=lateral

8. calyculus 0=absent, 1=vanilloid, 2=polystachyoid

9. slipper-shaped labellum 0=absent, 1=present

10. apiculate sepals 0=absent, 1=present

11. carinate petals 0=present, 1=absent

12. lip-column marginal adnation 0=absent, 1=present

13. dorsal median stamen 0=present, 1=absent

14. lateral inner stamens 0=present, 1=absent

15. anther orientation 0=erect, 1=bending late, 2=bending early

16. operculate anther 0=absent, 1=present

17. basal caudicles 0=absent, 1=present

18. hammer stipe 0=absent, 1=present

19. tegula 0=absent, 1=present

20. pollen unit 0=monad, 1=tetrad

21. massulae 0=absent, 1=orchidoid, 2=epidendroid, 3=arethusoid

22. pollinium texture 0=granular, 1=solid

23. pollinium number= 2 0=absent, 1=present

24. pollinium number= 8 0=absent, 1=longitudinal, 2=transverse

25. pollinium orientation 0=juxtaposed, 1=superposed

26. ovary locule number 0=one, 1=three

27. stigma 0=protruded, 1=sunken

28. viscidium 0=none, 1=diffuse, 2=detachable

29. Petal margins 0=entire or undulate, 1=denticulate

30. lip morphology 0=unlobed or trilobed with entire side-lobes, 1=trilobed with bilobed side-lobes

31. lip base 0=saccate, 1=spurred, 2=not saccate or spurred

32. lip appendages 0=absent, 1=present, at the entrance of sac or spur, 2=present, at the base or other part of the mid-lobe

33. column-foot 0=absent, 1=present

34. pollinium characters 0=cleft, 1=porate, 2=not cleft or porate

35. lip mid-lobe clawed at base 0=absent, 1=present

36. stipe 0=tapering, 1=oblong, 2=not tapering or oblong

37. spur 0=cylindrical, 1=horn-shaped, 2=not cylindrical or horn-shaped

38. lip colour 0=purple or purple markings, 1=white, 2=not purple and purple markings or white

39. stem 0=short, 1=elongate

40. plant clustered 0=present, 1=absent

41. caudicle 0=present, 1=absent
