# Supplementary material for: Paraholcoglossum and Tsiorchis, Two New Orchid Genera Established by Molecular and Morphological Analyses of the Holcoglossum Alliance
Source: PLoS One. 2011 Oct 10;6(10):e24864. doi: 10.1371/journal.pone.0024864 (PMC3189912; doi:10.1371/journal.pone.0024864)
Supplement: Table S1 — Pair-wise genetic distance of all species based on combined datasets of ITS, trnL-F, and matK. (DOC) [file pone.0024864.s024.doc]

**Table S1**. Pair-wise genetic distance of all species based on combined datasets of ITS, *trnL-F*, and *matK*.

|  |  | 1 | 2 | 3 | 4 | 5 | 6 | 7 | 8 | 9 | 10 | 11 | 12 | 13 | 14 | 15 | 16 | 17 | 18 | 19 | 20 | 21 | 22 | 23 | 24 | 25 | 26 | 27 | 28 | 29 | 30 |
| --- | --- | --- | --- | --- | --- | --- | --- | --- | --- | --- | --- | --- | --- | --- | --- | --- | --- | --- | --- | --- | --- | --- | --- | --- | --- | --- | --- | --- | --- | --- | --- |
| 1 | *Holcoglossum rupestre* |  |  |  |  |  |  |  |  |  |  |  |  |  |  |  |  |  |  |  |  |  |  |  |  |  |  |  |  |  |  |
| 2 | *H. flavescens* | 0.0039 |  |  |  |  |  |  |  |  |  |  |  |  |  |  |  |  |  |  |  |  |  |  |  |  |  |  |  |  |  |
| 3 | *H. sinicum* | 0.0039 | 0.0052 |  |  |  |  |  |  |  |  |  |  |  |  |  |  |  |  |  |  |  |  |  |  |  |  |  |  |  |  |
| 4 | *H. weixiense* | 0.0048 | 0.0061 | 0.0035 |  |  |  |  |  |  |  |  |  |  |  |  |  |  |  |  |  |  |  |  |  |  |  |  |  |  |  |
| 5 | *H. quasipinifolium* | 0.0153 | 0.0180 | 0.0126 | 0.0162 |  |  |  |  |  |  |  |  |  |  |  |  |  |  |  |  |  |  |  |  |  |  |  |  |  |  |
| 6 | *H. tsii* | 0.0048 | 0.0078 | 0.0078 | 0.0069 | 0.0140 |  |  |  |  |  |  |  |  |  |  |  |  |  |  |  |  |  |  |  |  |  |  |  |  |  |
| 7 | *H. lingulatum* | 0.0069 | 0.0100 | 0.0056 | 0.0083 | 0.0118 | 0.0048 |  |  |  |  |  |  |  |  |  |  |  |  |  |  |  |  |  |  |  |  |  |  |  |  |
| 8 | *H. omeiense* | 0.0069 | 0.0100 | 0.0056 | 0.0083 | 0.0118 | 0.0048 | 0.0000 |  |  |  |  |  |  |  |  |  |  |  |  |  |  |  |  |  |  |  |  |  |  |  |
| 9 | *H. linearifolium* | 0.0233 | 0.0256 | 0.0246 | 0.0246 | 0.0323 | 0.0224 | 0.0264 | 0.0264 |  |  |  |  |  |  |  |  |  |  |  |  |  |  |  |  |  |  |  |  |  |  |
| 10 | *H. nujiangense* | 0.0131 | 0.0144 | 0.0135 | 0.0135 | 0.0251 | 0.0140 | 0.0179 | 0.0179 | 0.0109 |  |  |  |  |  |  |  |  |  |  |  |  |  |  |  |  |  |  |  |  |  |
| 11 | *H. amesianum* | 0.0122 | 0.0144 | 0.0135 | 0.0140 | 0.0229 | 0.0135 | 0.0127 | 0.0127 | 0.0301 | 0.0215 |  |  |  |  |  |  |  |  |  |  |  |  |  |  |  |  |  |  |  |  |
| 12 | *H. auriculatum* | 0.0148 | 0.0171 | 0.0162 | 0.0170 | 0.0237 | 0.0166 | 0.0153 | 0.0153 | 0.0323 | 0.0246 | 0.0104 |  |  |  |  |  |  |  |  |  |  |  |  |  |  |  |  |  |  |  |
| 13 | *H. subulifolium* | 0.0083 | 0.0105 | 0.0096 | 0.0104 | 0.0219 | 0.0122 | 0.0109 | 0.0109 | 0.0301 | 0.0197 | 0.0056 | 0.0109 |  |  |  |  |  |  |  |  |  |  |  |  |  |  |  |  |  |  |
| 14 | *H. kimballianum* | 0.0131 | 0.0153 | 0.0144 | 0.0153 | 0.0224 | 0.0140 | 0.0127 | 0.0127 | 0.0296 | 0.0220 | 0.0078 | 0.0126 | 0.0091 |  |  |  |  |  |  |  |  |  |  |  |  |  |  |  |  |  |
| 15 | *H. wangii* | 0.0135 | 0.0157 | 0.0148 | 0.0157 | 0.0237 | 0.0153 | 0.0140 | 0.0140 | 0.0310 | 0.0224 | 0.0083 | 0.0131 | 0.0096 | 0.0030 |  |  |  |  |  |  |  |  |  |  |  |  |  |  |  |  |
| 16 | *Aerides flabellata* | 0.0219 | 0.0242 | 0.0233 | 0.0260 | 0.0327 | 0.0237 | 0.0224 | 0.0224 | 0.0405 | 0.0318 | 0.0229 | 0.0237 | 0.0215 | 0.0229 | 0.0233 |  |  |  |  |  |  |  |  |  |  |  |  |  |  |  |
| 17 | *A. krabiensis* | 0.0233 | 0.0264 | 0.0251 | 0.0260 | 0.0327 | 0.0251 | 0.0242 | 0.0242 | 0.0409 | 0.0341 | 0.0224 | 0.0233 | 0.0224 | 0.0233 | 0.0246 | 0.0292 |  |  |  |  |  |  |  |  |  |  |  |  |  |  |
| 18 | *A. odorata* | 0.0282 | 0.0305 | 0.0304 | 0.0323 | 0.0382 | 0.0291 | 0.0287 | 0.0287 | 0.0441 | 0.0373 | 0.0291 | 0.0277 | 0.0278 | 0.0287 | 0.0291 | 0.0166 | 0.0300 |  |  |  |  |  |  |  |  |  |  |  |  |  |
| 19 | *A. thibautiana* | 0.0323 | 0.0336 | 0.0336 | 0.0354 | 0.0418 | 0.0336 | 0.0332 | 0.0332 | 0.0488 | 0.0409 | 0.0296 | 0.0314 | 0.0291 | 0.0296 | 0.0300 | 0.0368 | 0.0273 | 0.0323 |  |  |  |  |  |  |  |  |  |  |  |  |
| 20 | *Papilionanthe biswasiana* | 0.0328 | 0.0359 | 0.0350 | 0.0359 | 0.0419 | 0.0337 | 0.0341 | 0.0341 | 0.0202 | 0.0273 | 0.0337 | 0.0265 | 0.0332 | 0.0328 | 0.0332 | 0.0428 | 0.0396 | 0.0423 | 0.0483 |  |  |  |  |  |  |  |  |  |  |  |
| 21 | *P. teres* | 0.0337 | 0.0359 | 0.0359 | 0.0368 | 0.0428 | 0.0346 | 0.0350 | 0.0350 | 0.0206 | 0.0282 | 0.0337 | 0.0300 | 0.0332 | 0.0328 | 0.0332 | 0.0410 | 0.0405 | 0.0405 | 0.0455 | 0.0100 |  |  |  |  |  |  |  |  |  |  |
| 22 | *Rhynchostylis retusa* | 0.0238 | 0.0269 | 0.0251 | 0.0260 | 0.0328 | 0.0256 | 0.0247 | 0.0247 | 0.0415 | 0.0337 | 0.0242 | 0.0251 | 0.0229 | 0.0233 | 0.0247 | 0.0310 | 0.0220 | 0.0328 | 0.0305 | 0.0424 | 0.0405 |  |  |  |  |  |  |  |  |  |
| 23 | *R. gigantea* | 0.0256 | 0.0278 | 0.0260 | 0.0278 | 0.0337 | 0.0274 | 0.0251 | 0.0251 | 0.0428 | 0.0360 | 0.0251 | 0.0265 | 0.0233 | 0.0247 | 0.0260 | 0.0296 | 0.0220 | 0.0300 | 0.0291 | 0.0428 | 0.0401 | 0.0184 |  |  |  |  |  |  |  |  |
| 24 | *Vanda brunnea* | 0.0175 | 0.0197 | 0.0197 | 0.0215 | 0.0282 | 0.0202 | 0.0197 | 0.0197 | 0.0341 | 0.0264 | 0.0175 | 0.0175 | 0.0162 | 0.0175 | 0.0180 | 0.0126 | 0.0251 | 0.0197 | 0.0327 | 0.0364 | 0.0355 | 0.0242 | 0.0256 |  |  |  |  |  |  |  |
| 25 | *V. coerulescens* | 0.0215 | 0.0238 | 0.0238 | 0.0255 | 0.0332 | 0.0242 | 0.0238 | 0.0238 | 0.0401 | 0.0314 | 0.0215 | 0.0215 | 0.0202 | 0.0215 | 0.0220 | 0.0131 | 0.0301 | 0.0219 | 0.0359 | 0.0405 | 0.0387 | 0.0301 | 0.0287 | 0.0100 |  |  |  |  |  |  |
| 26 | *V. pumila* | 0.0162 | 0.0193 | 0.0184 | 0.0201 | 0.0273 | 0.0188 | 0.0184 | 0.0184 | 0.0346 | 0.0260 | 0.0162 | 0.0166 | 0.0148 | 0.0153 | 0.0157 | 0.0148 | 0.0255 | 0.0224 | 0.0318 | 0.0360 | 0.0359 | 0.0256 | 0.0260 | 0.0083 | 0.0113 |  |  |  |  |  |
| 27 | *V. subconcolor* | 0.0166 | 0.0188 | 0.0188 | 0.0206 | 0.0282 | 0.0193 | 0.0189 | 0.0189 | 0.0350 | 0.0264 | 0.0166 | 0.0175 | 0.0153 | 0.0166 | 0.0171 | 0.0131 | 0.0251 | 0.0210 | 0.0327 | 0.0364 | 0.0355 | 0.0242 | 0.0256 | 0.0039 | 0.0087 | 0.0083 |  |  |  |  |
| 28 | *Ascocentrum ampullaceum* | 0.0211 | 0.0242 | 0.0224 | 0.0242 | 0.0319 | 0.0237 | 0.0224 | 0.0224 | 0.0396 | 0.0309 | 0.0211 | 0.0211 | 0.0197 | 0.0211 | 0.0215 | 0.0135 | 0.0296 | 0.0242 | 0.0364 | 0.0401 | 0.0401 | 0.0287 | 0.0301 | 0.0104 | 0.0126 | 0.0109 | 0.0109 |  |  |  |
| 29 | *Neofinetia falcata* | 0.0179 | 0.0211 | 0.0184 | 0.0211 | 0.0278 | 0.0206 | 0.0184 | 0.0184 | 0.0364 | 0.0278 | 0.0171 | 0.0180 | 0.0149 | 0.0171 | 0.0175 | 0.0197 | 0.0255 | 0.0264 | 0.0327 | 0.0369 | 0.0360 | 0.0242 | 0.0256 | 0.0122 | 0.0171 | 0.0131 | 0.0118 | 0.0175 |  |  |
| 30 | *Jumellea sagittata* | 0.0961 | 0.0987 | 0.0946 | 0.0976 | 0.1031 | 0.0976 | 0.0941 | 0.0941 | 0.1029 | 0.0974 | 0.0946 | 0.0946 | 0.0956 | 0.0926 | 0.0955 | 0.1011 | 0.0886 | 0.0964 | 0.0998 | 0.1013 | 0.0999 | 0.0957 | 0.0941 | 0.0946 | 0.1006 | 0.0965 | 0.0955 | 0.1006 | 0.0940 |  |
| 31 | *Microterangis hariotiana* | 0.0668 | 0.0702 | 0.0683 | 0.0702 | 0.0769 | 0.0683 | 0.0669 | 0.0669 | 0.0763 | 0.0715 | 0.0703 | 0.0708 | 0.0678 | 0.0674 | 0.0689 | 0.0702 | 0.0626 | 0.0682 | 0.0720 | 0.0769 | 0.0764 | 0.0675 | 0.0655 | 0.0683 | 0.0742 | 0.0688 | 0.0678 | 0.0722 | 0.0688 | 0.0784 |
