# Supplementary material for: Paraholcoglossum and Tsiorchis, Two New Orchid Genera Established by Molecular and Morphological Analyses of the Holcoglossum Alliance
Source: PLoS One. 2011 Oct 10;6(10):e24864. doi: 10.1371/journal.pone.0024864 (PMC3189912; doi:10.1371/journal.pone.0024864)
Supplement: Table S2 — Samples used in the gene sequencing and their information. (DOC) [file pone.0024864.s025.doc]

**Table S2. Samples used in the gene sequencing and their information.**

|  |  |  | ITS | | | | *trnL-F* | | | | *matK* | | |
| --- | --- | --- | --- | --- | --- | --- | --- | --- | --- | --- | --- | --- | --- |
| species | Locality  from China | Voucher  (NOCC) | Accession number | Sequence length | Length used in aligned |  | Accession number | Sequence length | Length used in aligned |  | Accession number | Sequence length | Length used in aligned |
| *Holcoglossum tsii* DL |  |  | EU558927 | 657 | 657 | | EU558902 | 870 | 870 | | AB217732 | 1691 | 1691 |
| *H. quasipinifolium* | Taiwan | Z. J. Liu2974 | HQ452909 | 773 | 657 | | HQ452939 | 1093 | 1093 | | HQ452924 | 1705 | 1705 |
| *H. rupestre* DL |  |  | ER558920 | 657 |  | | ER558885 | 870 |  | | ER558948 | 1612 |  |
| *H. rupestre* | Zhongdian | Z. J. Liu2658 | HQ452905 | 774 | 658 | | HQ452935 | 1108 | 1108 | | ER558948* | 1612* | 1612 |
| *H. flavescens* DL |  |  | EU558924 | 657 |  | | EU558898 | 910 |  | | EU558962 | 1612 |  |
| *H. flavescens* | Lijiang | Z. J. Liu2659 | HQ452904 | 773 | 657 | | HQ452934 | 1108 | 1108 | | HQ452919 | 1710 | 1710 |
| *H. weixiense* DL |  |  | EU558911 | 656 |  | | EU558889 | 870 |  | | EU5588957 | 1612 |  |
| *H. weixiense* | Weixi | Z. J. Liu2663 | HQ452900 | 773 | 658 | | HQ452930 |  | 1107 | | EU5588957* | 1612* | 1612 |
| *H. sinicum* DL |  |  | EU558919 | 660 |  | | EU558890 | 868 |  | | EU558956 | 1611 | 1611 |
| *H. sinicum* | Lijiang | Z. J. Liu2664 | HQ452906 | 775 | 659 | | HQ452936 | 1095 | 1095 | | EU558956* | 1611* |  |
| *H. nujiangense* DL |  |  | ER558910 | 656 |  | | ER558892 | 870 |  | | ER558959 | 1612 |  |
| *H. nujiangense* | Lushui | Z. J. Liu3498 | HQ452908 | 773 | 657 | | HQ452938 | 1096 | 1096 | | HQ452923 | 1703 | 1703 |
| *H. auriculatum* | Malipo | Z. J. Liu2758 | HQ452913 | 778 | 662 | | HQ452943 | 1117 | 1117 | | HQ452928 | 1691 | 1691 |
| *H. kimballianum* DL |  |  | ER558904 | 655 |  | | EUR558881 | 1056 |  | | EU558944 | 1611 |  |
| *H. kimballianum* | Malipo | Z. J. Liu2114 | JN106331 | 733 | 655 | | JN106338 | 1231 | 1221 | | JN106345 | 1769 | 1737 |
| *H. omeiense* DL |  |  | EU558908 | 658 |  | | EU558882 | 849 |  | | EU558950 | 1611 |  |
| *H. omeiense* | Emei | Z. J. Liu2652 | JN106332 | 694 | 659 | | JN106339 | 1072 | 1057 | | JN106346 | 1651 | 1717 |
| *H. wangii* DL |  |  | EU558903 | 655 |  | | EU558878 | 907 |  | | EU558945 | 1612 |  |
| *H. wangii* | Malipo | Z. J. Liu2818 | JN106333 | 730 | 655 | | JN106340 | 1128 | 1120 | | JN106347 | 1749 | 1718 |
| *H. lingulatum* DL |  |  | EU558907 | 658 |  | | EU558883 | 841 |  | | EU558949 | 1612 |  |
| *H. lingulatum* | Malipo | Z. J. Liu3544 | JN106334 | 736 | 655 | | JN106341 | 1092 | 1092 | | JN106348 | 1744 | 1717 |
| *H. subulifolium* DL |  |  | ER558905 | 657 |  | | EU558895 | 870 |  | | EU558951 | 1612 |  |
| *H. subulifolium* | Hainan | Z. J. Liu3249 | JN106335 | 700 | 657 | | JN106342 | 883 | 798 | | JN106349 | 1768 | 1729 |
| *H. amesianum* DL |  |  | EU558906 | 656 |  | | EU558879 | 899 |  | | EU558946 | 1612 |  |
| *H. amesianum* | Puer | Z. J. Liu2716 | JN106336 | 691 | 656 | | JN106343 | 1117 | 1117 | | JN106350 | 1769 | 1730 |
| *H. linearifolium* | Malipo | Z. J. Liu4865 | JN106337 | 774 | 658 | | JN106344 | 1096 | 1096 | | JN106351 | 1757 | 1726 |
| *Papilionanthe biswasiana* | Simao | Z. J. Liu4815 | HQ452914 |  |  | | HQ452944 |  |  | | HQ452929 |  |  |
| [*P. teres*](http://www.ncbi.nlm.nih.gov/Taxonomy/Browser/wwwtax.cgi?id=339107) DL | | | EU558934 |  | | | EU558872 | | | | EU558937 | | |
| [*Rhynchostylis retusa*](http://www.ncbi.nlm.nih.gov/Taxonomy/Browser/wwwtax.cgi?id=257352) DL | | | EU558933 |  | | | EU558873 | | | | EU558938 | | |
| [*R. gigantea*](http://www.ncbi.nlm.nih.gov/Taxonomy/Browser/wwwtax.cgi?id=280450) DL | | | AY912264 |  | | | DQ194989^†^ & DQ195006^†^ | | | | AY557202^†^ | | |
| [*Vanda brunnea*](http://www.ncbi.nlm.nih.gov/Taxonomy/Browser/wwwtax.cgi?id=536165) DL | | | EU558932 |  | | | EU558874 | | | | EU55893 | | |
| [*V. coerulescens*](http://www.ncbi.nlm.nih.gov/Taxonomy/Browser/wwwtax.cgi?id=312812) DL | | | EU558931 |  | | | EU558875 | | | | EU558940 | | |
| [*V. pumila*](http://www.ncbi.nlm.nih.gov/Taxonomy/Browser/wwwtax.cgi?id=331261) DL | | | EU558930 |  | | | EU558876^†^ | | | | EU558941 | | |
| [*V. subconcolor*](http://www.ncbi.nlm.nih.gov/Taxonomy/Browser/wwwtax.cgi?id=536166) DL | | | EU558929 |  | | | EU558877^†^ | | | | EU558942 | | |
| [*Aerides flabellata*](http://www.ncbi.nlm.nih.gov/Taxonomy/Browser/wwwtax.cgi?id=331161) DL | | | AB217528 |  | | | EU558870 | | | | AB217704 | | |
| [*A. odorata*](http://www.ncbi.nlm.nih.gov/Taxonomy/Browser/wwwtax.cgi?id=331162) DL | | | AB217529 |  | | | EF670389 | | | | AB217705^†^ | | |
| *A. krabiensis* DL | | | EF670341 |  | | | EF670404^†^ | | | | EF655784 | | |
| *A. thibautiana* DL | | | EF670337 |  | | | EF670398 | | | | EF655813 | | |
| [*Ascocentrum ampullaceum*](http://www.ncbi.nlm.nih.gov/Taxonomy/Browser/wwwtax.cgi?id=312809) DL | | | AY912260 |  | | | DQ194985^†^ & DQ195000^†^ | | | | EU558935 | | |
| [*Neofinetia falcata*](http://www.ncbi.nlm.nih.gov/Taxonomy/Browser/wwwtax.cgi?id=78812) DL | | | AY912262 |  | | | DQ091442 | | | | EF655782 | | |
| [*Microterangis hariotiana*](http://www.ncbi.nlm.nih.gov/Taxonomy/Browser/wwwtax.cgi?id=331149) DL | | | AB217523 |  | | | DQ091467 | | | | AB217699 | | |
| [*Jumellea sagittata*](http://www.ncbi.nlm.nih.gov/Taxonomy/Browser/wwwtax.cgi?id=331151) DL | | | AB217522 |  | | | DQ091555 | | | | AB217698 | | |

Notes: the species with DL are accessed from GenBank; *, there are fragments of the *matK* that we could not get and accessed from GenBank; †, the sequences miss some partial characters.
