# Supplementary material for: Paraholcoglossum and Tsiorchis, Two New Orchid Genera Established by Molecular and Morphological Analyses of the Holcoglossum Alliance
Source: PLoS One. 2011 Oct 10;6(10):e24864. doi: 10.1371/journal.pone.0024864 (PMC3189912; doi:10.1371/journal.pone.0024864)
Supplement: Table S3 — Genetic distances between sections of the Holcoglossum clade. (DOC) [file pone.0024864.s026.doc]

**Table S3.** Genetic distances between sections of the *Holcoglossum* clade*.*

|  | **1** | **2** | **3** |
| --- | --- | --- | --- |
| 1. *H.* sect. *Holcoglossum* | － |  |  |
| 2. *H.* sect. *Sorotylos* | 0.0105 | － |  |
| 3. *H.* sect. *Nujiangensia* | 0.0202 | 0.0241 | － |
