# Supplementary material for: Paraholcoglossum and Tsiorchis, Two New Orchid Genera Established by Molecular and Morphological Analyses of the Holcoglossum Alliance
Source: PLoS One. 2011 Oct 10;6(10):e24864. doi: 10.1371/journal.pone.0024864 (PMC3189912; doi:10.1371/journal.pone.0024864)
Supplement: Table S4 — Morphological data matrix for the phylogenetic analysis. (DOC) [file pone.0024864.s027.doc]

**Table S4. Morphological data matrix for the phylogenetic analysis.**

| **Taxa** | **Characters** |
| --- | --- |
|  | 11111111112222222222333333333344  12345678901234567890123456789012345678901 |
| *Aerides flabellata* | 01101110001001210101011000120012000000111 |
| *Aerides krabiensis* | 01101110001001210101011000120011100000111 |
| *Aerides odorata* | 01101110001001210101011000120010100000111 |
| *Aerides thibautiana* | 0110111000100121010101100012001?100000111 |
| *Ascocentrum ampullaceum* | 01101110001001210101011000120011010002111 |
| *Holcoglossum amesianum* | 01121110000001210101011000120001111122111 |
| *Holcoglossum auriculatum* | 01111110000001210101011000121001111111111 |
| *Holcoglossum flavescens* | 00111110000001210101011000120012110011001 |
| *Holcoglossum kimballianum* | 01111110000001210101011000120012100000110 |
| *Holcoglossum linearifolium* | 00111110000001210101011000120012110011001 |
| *Holcoglossum lingulatum* | 00111110000001210101011000120112110011001 |
| *Holcoglossum nujiangense* | 00111110000001210101011000120012110011001 |
| *Holcoglossum omeiense* | 00111110000001210101011000120112110011001 |
| *Holcoglossum quasipinifolium* | 00111110000001210101011000120012110011001 |
| *Holcoglossum rupestre* | 00111110000001210101011000120012110011001 |
| *Holcoglossum sinicum* | 00111110000001210101011000120012110011001 |
| *Holcoglossum subulifolium* | 01111110000001210101011000120001111111111 |
| *Holcoglossum tsii* | 00111110000001210101011000120012110011001 |
| *Holcoglossum wangii* | 01111110000001210101011000120012100000110 |
| *Holcoglossum weixiense* | 00111110000001210101011000120012110011001 |
| *Jumellea sagittata* | 01101110001001210101011000120010111201111 |
| *Microterangis hariotiana* | 01101110001001210101011000120010020000111 |
| *Neofinetia falcata* | 01101110001001210101011000120012000000001 |
| *Papilionanthe biswasiana* | 01111110001001210101011000120011100110111 |
| *Papilionanthe teres* | 01111110001001210101011000120010100000111 |
| *Rhynchostylis retusa* | 01101110001001210101011000120010100000110 |
| *Rhynchostylis gigantea* | 01101110001001210101011000120010100000110 |
| *Vanda brunnea* | 01101110001001210101011000120012000000111 |
| *Vanda coerulescens* | 01101110001001210101011000120012000000111 |
| *Vanda pumila* | 01101110001001210101011000120010000000111 |
| *Vanda subconcolor* | 01101110001001210101011000120010000000111 |
